# Genital Mycoplasmas and Biomarkers of Inflammation and Their Association With Spontaneous Preterm Birth and Preterm Prelabor Rupture of Membranes: A Systematic Review and Meta-Analysis

**DOI:** 10.3389/fmicb.2022.859732

**Published:** 2022-03-30

**Authors:** Nathalia M. Noda-Nicolau, Ourlad Alzeus G. Tantengco, Jossimara Polettini, Mariana C. Silva, Giovana F. C. Bento, Geovanna C. Cursino, Camila Marconi, Ronald F. Lamont, Brandie D. Taylor, Márcia G. Silva, Daniel Jupiter, Ramkumar Menon

**Affiliations:** ^1^Department of Pathology, Botucatu Medical School, Universidade Estadual Paulista, Botucatu, Brazil; ^2^Division of Basic and Translational Research, Department of Obstetrics and Gynecology, The University of Texas Medical Branch at Galveston, Galveston, TX, United States; ^3^Department of Biochemistry and Molecular Biology, College of Medicine, University of the Philippines Manila, Manila, Philippines; ^4^Graduate Program in Biomedical Sciences, Universidade Federal da Fronteira Sul, Passo Fundo, Brazil; ^5^Department of Basic Pathology, Setor de Ciências Biológicas, Universidade Federal do Paraná, Curitiba, Brazil; ^6^Research Unit of Gynaecology and Obstetrics, Department of Gynecology and Obstetrics, Institute of Clinical Research, University of Southern Denmark, Odense, Denmark; ^7^Division of Surgery, Northwick Park Institute for Medical Research, University College London, London, United Kingdom; ^8^Department of Preventive Medicine and Community Health, University of Texas Medical Branch, Galveston, TX, United States

**Keywords:** inflammatory cytokines, *Mycoplasma* species, pregnancy, preterm birth, *Ureaplasma* species, pregnancy adverse outcomes

## Abstract

Genital mycoplasmas (GM), such as *Mycoplasma hominis, Mycoplasma genitalium, Ureaplasma parvum*, and *Ureaplasma urealyticum* are commonly associated with spontaneous preterm labor (SPTL), spontaneous preterm birth (PTB), and preterm prelabor rupture of membranes (PPROM). This study determined the association between GM and such adverse pregnancy outcomes. We searched for studies published 1980–2019 in MEDLINE, EMBASE, and Web of Science. Studies were eligible when GM was detected during pregnancy. We included 93 and 51 studies in determining the prevalence and the inflammatory biomarkers associated with GM, respectively, using the “metafor” package within R. The protocol was registered with PROSPERO (registration no. CRD42016047297). Women with the studied adverse pregnancy outcomes had significantly higher odds of presence with GM compared to women who delivered at term. For PTB, the odds ratios were: *M. hominis* (OR: 2.25; CI: 1.35–3.75; *I*^2^: 44%), *M. genitalium* (OR: 2.04; CIL 1.18–3.53; *I*^2^: 20%), *U. parvum* (OR: 1.75; CI: 1.47–2.07; *I*^2^: 0%), *U. urealyticum* (OR: 1.50; CI: 1.08–2.07; *I*^2^: 58%). SPTL had significantly higher odds with *M. hominis* (OR: 1.96; CI: 1.19–3.23; *I*^2^: 1%) or *U. urealyticum* (OR: 2.37; CI: 1.20–4.70; *I*^2^: 76%) compared to women without SPTL. Women with PPROM had significantly higher odds with *M. hominis* (OR: 2.09; CI: 1.42–3.08; *I*^2^: 0%) than women without PPROM. However, our subgroup analysis based on the diagnostic test and the sample used for detecting GM showed a higher prevalence of GM in maternal samples than in fetal samples. GM presence of the cervix and vagina was associated with lower odds of PTB and preterm labor (PTL). In contrast, GM presence in the AF, fetal membrane, and placenta was associated with increased odds of PTB and PTL. However, genital mycoplasmas may not elicit the massive inflammation required to trigger PTB. In conclusion, GM presence in the fetal tissues was associated with significantly increased odds of PTB and PTL.

## Introduction

The onset of labor before 37 weeks of gestation, defined as spontaneous preterm labor (SPTL), that may leads to preterm birth (PTB), along with the preterm prelabor rupture of the membranes (PPROM), characterized by the rupture of membranes before labor that occurs before 37 weeks of pregnancy, are two major complications of pregnancies affecting ∼11% of all pregnancies worldwide (Chawanpaiboon et al., 2019).

Microbial invasion of the amniotic cavity (MIAC) followed by intraamniotic inflammation is associated with most cases of SPTL, PTB, and PPROM. Approximately 50% of all PTBs, defined as births that occurs before 37 weeks of gestation, and 20–40% of PPROM have an infectious and inflammatory association (Jacobsson et al., 2003; Menon and Fortunato, 2007; Romero et al., 2015). Furthermore, MIAC and intraamniotic infection and inflammation are associated with histologic chorioamnionitis, funisitis, and systemic fetal inflammatory response during pregnancy (Romero et al., 2011; Kacerovsky et al., 2013b). MIAC is often detected as a single bacterial infection (Stranik et al., 2021). However, polymicrobial bacterial are documented in the amniotic cavity and associated with poor perinatal prognosis in preterm labor (PTL) (Yoneda et al., 2016). In this context, the most common of which are *Ureaplasma* and *Mycoplasma*, collectively known as genital mycoplasmas (Taylor-Robinson and Lamont, 2010; Tantengco and Yanagihara, 2019). Conversely, the literature on the use of prophylactic antibiotics to prevent PTB is confusing (Lamont, 2015). The use of antibiotics has not successfully reduced global rates of PTB or PPROM. Ambiguity concerning the effectiveness of antibiotic interventions and reducing the risk of PTB and PPROM can be attributed to several factors (Oh et al., 2019; Tanaka et al., 2019; Kacerovsky et al., 2020): (i) although there is an association between MIAC and/or intraamniotic inflammation with PTB and PPROM, an infectious etiology and the kinetics of intraamniotic infection and inflammation is difficult to determine; (ii) microbial load, polymicrobial nature and sites of microbial localization are critical in determining mechanistic events that can lead to PTB or PPROM; (iii) various underlying pathologies associated with feto-maternal uterine tissues that can compromise both antimicrobial and innate immune defense can cause MIAC, which might be a secondary condition; (iv) inflammation and inflammatory mediators generated in response to MIAC are not homogeneous.

Mechanistic models have not helped the derivation of management strategies because they may be indefinite in determining the contribution of pathogens to the pathways of PTB and PPROM. Our *in vitro* models have shown that the fetal inflammatory response differs based on polymicrobial combinations and their respective load (Noda-Nicolau et al., 2016; Polettini et al., 2018). Combinations of *Gardnerella vaginalis* and genital mycoplasmas produced distinct and load-dependent inflammatory profiles. A combination of high loads of *G. vaginalis* and a low load of genital mycoplasmas caused an immense fetal inflammatory response (Noda-Nicolau et al., 2016). Conversely, a high mycoplasma load and low *G. vaginalis* load produced a balanced inflammatory response with a robust anti-inflammatory response. Therefore, genital mycoplasmas at various loads and combinations are less proinflammatory than *G. vaginalis* (Noda-Nicolau et al., 2016).

Nonetheless, other studies have shown that *Ureaplasma parvum* can cause maternal T cell activation (Friedland et al., 2016). *In vivo*, animal models have shown that vaginal or intraamniotic infection inoculation of mycoplasmas can trigger SPTL (Motomura et al., 2020; Pavlidis et al., 2020). All these models have indicated several pathophysiologic aspects of PTB induced by microbes. However, these models have limitations and do not accurately mimic MIAC or intraamniotic inflammation seen in humans. These studies can be improved, and better mechanistic information can be generated if we have better knowledge from clinical studies concerning the prevalence of infection, infectious agents, and data on inflammatory mediators correlated with infection.

To synthesize knowledge and improve our understanding of the role of genital mycoplasmas in MIAC and intraamniotic inflammation contributing to SPTL, PTB, and PPROM, a comprehensive systematic review (SR) and metanalysis (MA) was conducted. Fetal, neonatal, and postpartum maternal complications due to these microorganisms were not a part of this study. This study determined the prevalence of genital mycoplasma presence in PTB and PPROM. We also checked any association between the genital mycoplasmas and SPTL, PTB, and PPROM. Lastly, we evaluated the potential biomarkers associated with genital mycoplasma presence in SPTL, PTB, and PPROM.

## Materials and Methods

### Eligibility Criteria, Information Sources, Search Strategy

An SR was conducted according to the PRISMA statement (Liberati et al., 2009) and performed using three biomedical literature databases, MEDLINE, EMBASE, and Web of Science, selecting relevant studies concerning mycoplasma presence and preterm labor regardless of delivery at term or preterm (SPTL), SPTL followed by PTB (PTB), or preterm prelabor rupture of the membranes (PPROM). We sought studies between 1980 and 2019 using the following terms: *Ureaplasma urealyticum* OR *U. parvum* OR *Mycoplasma hominis* OR *Mycoplasma genitalium* AND pregnancy. This study was supported by the Strategic Research Commitment fund from the University of Texas Medical Branch at Galveston, UTMB, and 1R01HD100729 (NIH/NICHD) to R Menon.

### Study Selection

Case-control, cross-sectional, prospective, and retrospective cohort studies were considered eligible in this SR, presenting the following criteria: subjects were pregnant women with the target outcomes either SPTL that leads to preterm birth (PTB) or PPROM; detection of genital mycoplasma presence in either maternal or fetal biological compartments during pregnancy. Cross-sectional studies were used in estimating the pooled prevalence of genital mycoplasma among patients with PTL, PTB, and PPROM. On the other hand, case-control and cohort studies were used in determining the association between genital mycoplasma and PTL, PTB, and PPROM.

Reviews, editorials, letters, case reports, conferences, summaries, books, opinions, *in vitro* studies, studies of diagnostics methodologies, and studies in animal models were excluded, and a search for unpublished studies was not performed. Other characteristics of excluded studies were maternal exposure to infection(s) caused by genital mycoplasmas during pregnancy was not directly measured but mentioned as a possible pathogen; infection by genital mycoplasmas in term infants; only term deliveries; PTB caused by other associated complications, such as preeclampsia, intrauterine growth restriction, stillbirth, diabetes, placental abruption, multiple pregnancies; the full-text of the study was not written in English, Portuguese, Spanish or French.

### Data Extraction

Two authors (NN-N and MGS) independently performed the screening of the records, first by titles and then by abstracts. Full-text analysis was performed by seven authors (NN-N, JP, OT, MCS, GB, GC, and CM) for articles that fulfilled the predetermined selection criteria for this SR. Discrepancies between the reviewers were discussed until consensus was reached. For each included study, we extracted the following primary data: author’s names, year of publication, type of study, the country where the study was conducted, study objective and design; the total number of participants, the pregnancy outcome, number of cases and controls (when applicable), gestational age or trimester at the time of sample collection, source of the biological sample, species of genital Mycoplasma detected, methodology used to identify genital mycoplasmas, the method for detecting inflammatory biomarkers, type of samples used for biomarker analysis and analytical approaches. The data collected were compared and discussed by the reviewers, and disagreements were resolved by consensus.

### Assessment of Risk of Bias

Six authors (NN-N, JP, OT, MCS, GB, and GC) assessed the quality of each included study, which was evaluated according to a set of parameters defined for this review using the Newcastle Ottawa Scale for case-control, cohort, and cross-sectional studies (Wells et al., 2013; Lo et al., 2014). The criteria for quality assessment are shown in Supplementary Tables 1–3.

### Data Synthesis and Statistical Analysis

A meta-analysis (MA) was carried out using random-effects models to determine the prevalence of genital mycoplasma with SPTL, PTB, and PPROM, considering all types of studies. For association analysis, only case-control and cohort studies were included. Forest and funnel plots were produced. A *p*-value of <0.05 was considered statistically significant. Higgins *I*^2^ was used to assess heterogeneity with *I*^2^ value of >50% regarded as high heterogeneity. All analyses were performed using the “metafor” package within R (R Core Team, 2020).

## Results

### Study Selection

**FIGURE 1:**
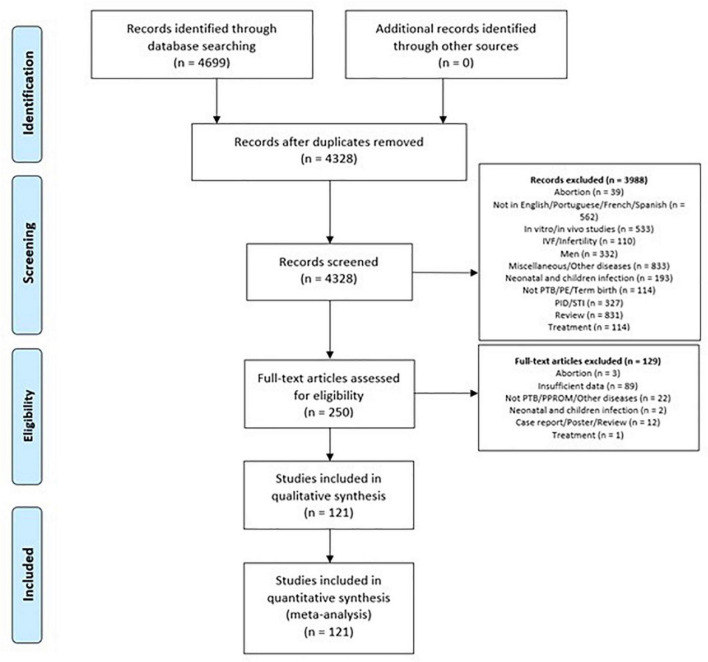
Preferred reporting items for systematic reviews and meta-analyses flow diagram.

We identified 4699 articles, and after the screening process, we retained 121 articles and included these in the qualitative synthesis of this SR (Figure 1). This included 93 articles showing the prevalence and association of genital mycoplasma and adverse pregnancy outcomes, including SPTL, PTB, and PROM, and 51 articles reporting the levels of inflammatory biomarkers in pregnant women with genital mycoplasma and women with any of these adverse pregnancy outcomes. Excluded studies are described in Supplementary Table 4.

### Study Characteristics

#### Genital Mycoplasma and Adverse Pregnancy Outcomes

Of the 93 articles that reported genital mycoplasma presence associated with SPTL, PTB, and PPROM, 61 were cross-sectional studies, while 32 were case-control and cohort studies (Supplementary Figure 1). Of the cross-sectional studies, 23 had PTB as an outcome, 28 on SPTL, and 27 on PPROM. Among the case-control and cohort studies, 24 studies had PTB as the primary outcome, ten on SPTL, and only two on PPROM. Specific details of the included studies are shown in Supplementary Tables 5, 6.

**FIGURE 2:**
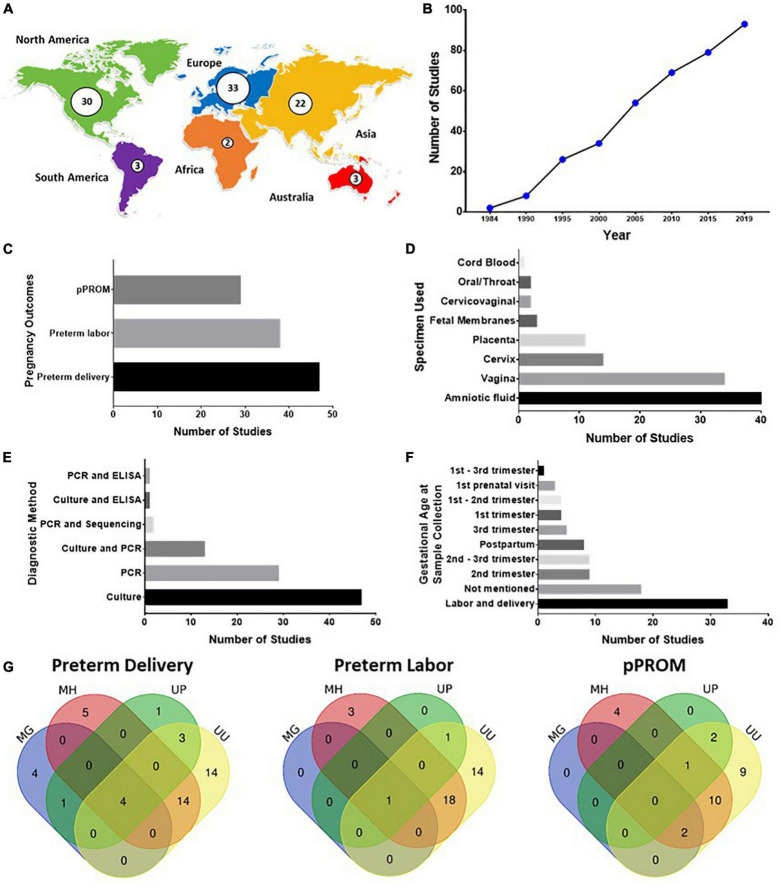
**(A–G)** Summary of studies on the association of genital Mycoplasma infection with preterm birth. **(A)** Geographical distribution of published studies on genital Mycoplasma and preterm birth. **(B)** Cumulative count of published studies in genital Mycoplasma and preterm birth. **(C)** Types of pregnancy outcomes studied. **(D)** Samples used for diagnosing genital *Mycoplasma* infections **(E)** Diagnostic method used for detecting genital Mycoplasma infections **(F)** Gestational age of patients during the time of sample collection for diagnosing genital Mycoplasma infections. **(G)** Venn diagrams depicting the combination of different genital *Mycoplasma* species detected from patients with spontaneous preterm labor (SPTL), preterm birth (PTB), and preterm prelabor rupture of membranes (PPROM).

Most of the studies on the association of genital mycoplasma presence with adverse pregnancy outcomes were from Europe (33/93), North America (30/93), and Asia (22/93). There were few studies from Africa (2/93), Australia (3/93), and South America (3/93) (Figure 2A and Supplementary Tables 5, 6). There was a continuous increase in published articles on this topic from 1984 to the present (Figure 2B). PTB is the most common pregnancy outcome associated with genital mycoplasma presence (47/93), followed by SPTL (38/93) and PPROM (29/93) (Figure 2C and Supplementary Tables 5, 6). The samples used and the diagnostic test to detect genital mycoplasma presence varied across different studies. The most used sample was amniotic fluid (AF) (40/93) and vaginal samples (34/93), while culture (47/93) and nucleic acid amplification test (NAAT)/polymerase chain reaction (PCR) test (29/93) were the most common diagnostic method used for genital mycoplasma diagnosis (Figures 2D,E and Supplementary Tables 5, 6). The time of collection of these samples varied between studies, given that the most common time of collection was at the time of labor and delivery (33/93). Still, other studies collected samples as early as the first trimester (9/93), while some studies collected samples postpartum (8/93) (Figure 2F and Supplementary Tables 5, 6). This difference in the timing of sample collection may have biased the associations made between genital mycoplasma presence and adverse pregnancy outcomes.

Most of the included studies in this SR only detected one species of genital mycoplasma with *U. urealyticum* being the most studied, followed by *M. hominis*. There were fewer studies that focused on *M. genitalium* and *U. parvum*. Several studies detected co-infection of different species of genital mycoplasmas, mainly *U. urealyticum* and *M. hominis* co-infection (Figure 2G). Further details of the included studies are shown in Supplementary Tables 5, 6.

#### Genital Mycoplasma and Inflammatory Biomarkers

**FIGURE 3:**
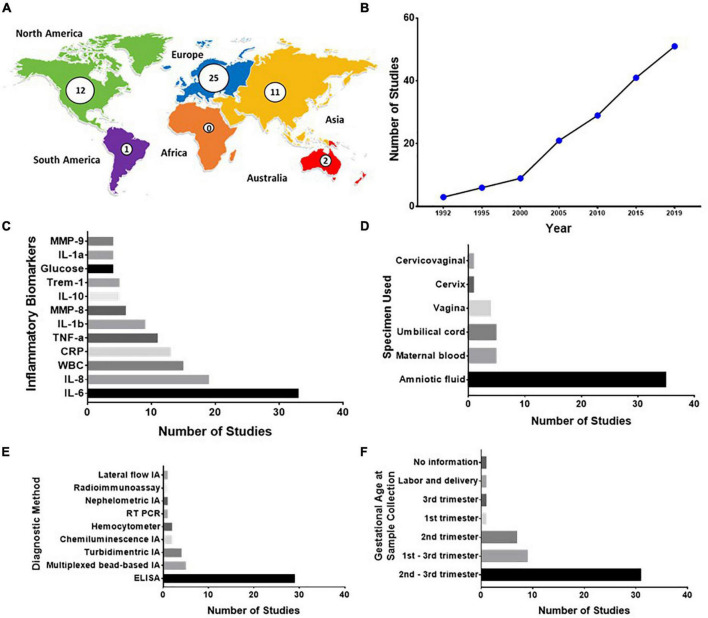
**(A–F)** Summary of studies on the levels of inflammatory biomarker from pregnant patients with genital Mycoplasma infections. **(A)** Geographical distribution of published studies on the association between inflammation and genital Mycoplasma infections during pregnancy and preterm birth. **(B)** Cumulative count of published studies. **(C)** Top ten most detected inflammatory biomarkers. **(D)** Samples used for diagnosing genital Mycoplasma infections **(E)** Diagnostic method used for detecting the inflammatory biomarkers from pregnant patients with genital Mycoplasma infections. **(F)** Gestational age of patients during the time of sample collection.

The studies on the levels of the inflammatory biomarker among pregnant women with genital mycoplasma presence were mainly from Europe (25/51), North America (12/51), and Asia (11/51). There were only two studies from Australia, one from South America, and none from Africa (Figure 3A and Supplementary Tables 7, 8). The number of studies in this field progressively increased since the first publication in 1992 (Figure 3B). Most of the inflammatory biomarkers detected in the studies included in this SR were proinflammatory cytokines such as IL-6, IL-8, C-reactive protein (CRP), tumor necrosis factor (TNF)-α, IL-1β, matrix metalloproteinase (MMP)-8, triggering receptor expressed on myeloid cells (TREM)-1, IL-1α, and MMP-9. IL-10 was the only anti-inflammatory cytokine included in the panel of inflammatory biomarkers detected in four studies included in this SR (Jacobsson et al., 2009; Kacerovsky et al., 2012, 2013a; Payne et al., 2014; Figure 3C). These biomarkers were collected from different samples, including AF (35/51), maternal blood (5/51), and umbilical cord blood samples (5/51) (Figure 3D). ELISA was the most used assay in detecting inflammatory biomarkers (Figure 3E), and the samples used for detecting these biomarkers were usually collected during the 2nd and 3rd trimester of pregnancy (Figure 3F). Most of the studies only collected single samples and no follow-up samples. Further details of the included studies are shown in Supplementary Tables 7, 8.

We performed a MA to determine the levels of different inflammatory biomarkers among pregnant women with and without genital mycoplasma presence. However, MA was not feasible due to the lack of sufficient studies that could be combined. Factors included heterogeneity in the set of inflammatory biomarkers, the diagnostic method used and reporting of inflammatory biomarker levels. There were also considerable differences in the type of samples used and the time of collection. Accordingly, we created a qualitative synthesis table (Supplementary Tables 7, 8) listing all the inflammatory biomarkers detected in pregnant patients with genital mycoplasma colonization.

We observed that pregnant women who experienced SPTL, PTB, and PPROM had significantly higher inflammatory biomarkers than those with term delivery (Supplementary Table 7). Specifically, cervicovaginal fluid concentrations of IL-1α, IL-1β, IL-6, RANTES, and TNFr1 and AF concentration of IL-6, IL-8, MMP-8, and TNFα were elevated in women with PTB. For women in SPTL, cervicovaginal fluid concentrations of IL-6 and IL-8 and AF levels of IL-1α, IL-1β, IL-6, and IL-8 were significantly elevated compared to women who gave birth at term. Women with PPROM had substantially higher levels of CRP in maternal serum, IL-6 and IL-8 in the vaginal fluid, IL-6, IL-8, and TNFα in cord blood samples, and C-C Motif Chemokine Ligand (CCL)-2 gene, CCL-3 gene, CRP, intercellular adhesion molecules (ICAM)-1, IL-6, IL-8, IL-10, MMP-8, and TNFα in the AF (Supplementary Table 7).

Among those women with adverse pregnancy outcomes, we also observed detection of *Mycoplasma* spp. or *Ureaplasma* spp. during pregnancy was associated with a significant increase in several proinflammatory biomarkers, including CRP, IL-6, IL-8, MMP-8, MMP-9, and TREM-1. However, one study also reported a significant increase in IL-10, an anti-inflammatory cytokine (Kacerovsky et al., 2013a). We then looked at studies reporting the inflammatory biomarker levels associated with each species of genital mycoplasmas. There were no studies on the concentration of inflammatory biomarkers in pregnant women infected with *M. genitalium*. Several studies reported significantly higher levels of IL-1β, IL-4, IL-6, and IL-16 from the AF samples of pregnant women diagnosed with *M. hominis* presence than uninfected women. One study also reported a positive correlation between *U. parvum* load and IL-8 levels from the AF samples of pregnant women who delivered preterm (Kasper et al., 2010). Conversely, *U. urealyticum* presence among pregnant women was also associated with significantly higher AF levels of IL-1β, IL-6, MMP-8, and TNFα compared to uninfected pregnant women (Supplementary Table 8).

### Risk of Bias of Included Studies

Regarding the quality of the included studies, we observed that most of the cross-sectional studies were assessed as satisfactory (17/74), good (33/74), and very good quality papers (20/74). Only four studies were evaluated as unsatisfactory due to low sample size, lack of description on the assessment of pregnancy outcomes, and the analysis did not control for confounding factors. For cohort and case-control studies, most of the papers were assessed as satisfactory (4/47), good (30/47), or very good quality (12/47). Only one paper was evaluated as unsatisfactory due to the low sample size and lack of operational definitions of primary outcomes. However, those unsatisfactory papers were retained in the analysis, as their results regarding mycoplasma prevalence or biomarkers did not influence our study (Supplementary Tables 1–3).

### Synthesis of Results

#### The Pooled Prevalence of Genital Mycoplasma Presence

**FIGURE 4:**
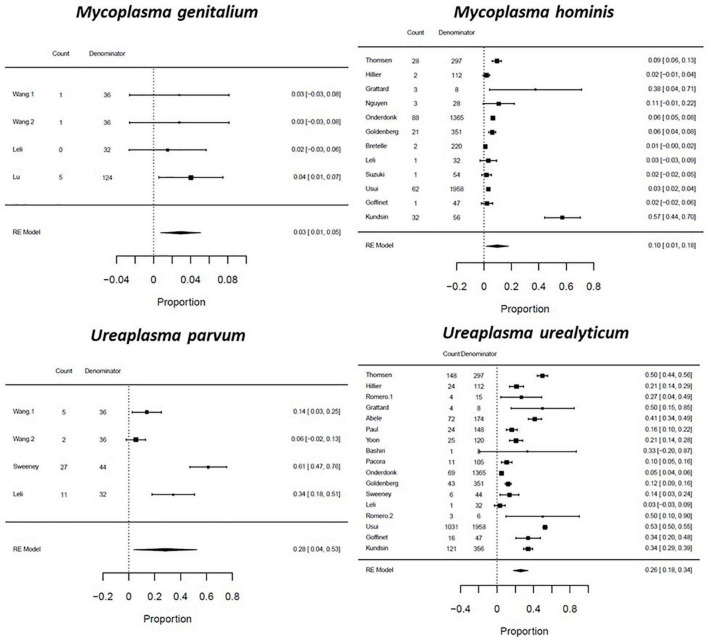
Forest plot for the pooled proportion of genital Mycoplasma infection among preterm birth patients.

**FIGURE 5:**
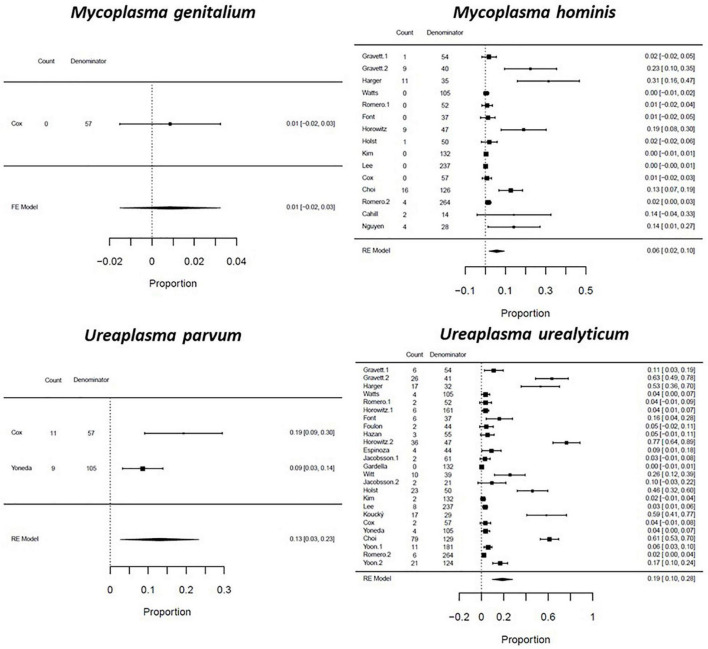
Forest plot for the pooled proportion of genital Mycoplasma infection among spontaneous preterm labor patients.

**FIGURE 6:**
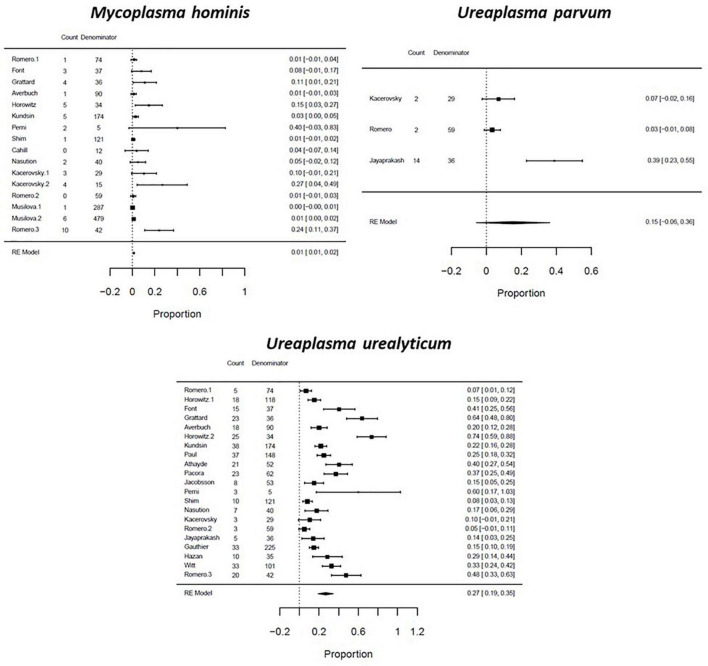
Forest plot for the pooled proportion of genital Mycoplasma infection among preterm prelabor rupture of membranes (PPROM) patients.

The pooled prevalence of genital mycoplasma presence are reported by pregnancy outcome: among women with PTB, *U. parvum* had the highest prevalence of 0.28 (CI: 0.04–0.53; *I*^2^: 94%), followed by *U. urealyticum* with a proportion of 0.26 (CI: 0.18–0.34; *I*^2^: 98%), *M. hominis* at 0.10 (CI: 0.01–0.18; *I*^2^: 99%), and *M. genitalium* at 0.03 (CI: 0.01–0.05; *I*^2^: 0%) (Figure 4). Among women with SPTL, *U. urealyticum* was the most common with a proportion of 0.19 (CI: 0.10–0.28; *I*^2^: 99%), followed by *U. parvum* (0.13; CI: 0.03–0.23; *I*^2^: 70%), *M. hominis* (0.06; CI: 0.02–0.10; *I*^2^: 98%), and *M. genitalium* (0.01; CI: -0.02–0.03; *I*^2^: 0%) (Figure 5). For women with PPROM, *U. urealyticum* was also the most prevalent with a proportion of 0.27 (CI: 0.19–0.35; *I*^2^: 94%), followed by *U. parvum* (0.15; CI: -0.06–0.36; *I*^2^: 94%), and *M. hominis* (0.01; CI: 0.01–0.02; *I*^2^: 27%). There were no studies that reported on the prevalence of *M. genitalium* among women with PPROM (Figure 6).

We then performed a subgroup analysis based on the type of sample used, and the methods used to detect genital mycoplasma. We observed differences in the prevalence of genital mycoplasmas based on the diagnostic methods used. *U. parvum* had a higher prevalence in studies that used PCR tests, while *U. urealyticum* had a higher prevalence in studies that used the culture method for detection (Supplementary Table 9). We also observed that most genital mycoplasmas had a higher pooled prevalence in studies that used maternal samples such as cervical and vaginal samples compared to studies that used fetal samples such as amniotic fluid, fetal membrane, and placenta (Supplementary Table 10).

To determine the prevalence of polymicrobial presence among women with adverse pregnancy outcomes, we retrieved all articles that reported co-infection with at least two species of genital mycoplasmas. We have presented a qualitative synthesis of data in Supplementary Table 11. Only co-infection with *M. hominis* and *U. urealyticum* were reported in previous studies. For women with PTB, the reported prevalence was 0.05 (Goldenberg et al., 2008). Based on seven studies, the proportion of *M. hominis* and *U. urealyticum* co-infection ranged from 0 to 0.19 (Gravett et al., 1986; Romero et al., 1992b; Watts et al., 1992; Horowitz et al., 1995; Holst et al., 2005; Kim et al., 2012; Lee et al., 2013). Conversely, the proportion of *M. hominis* and *U. urealyticum* co-infection among women with PPROM ranged from 0.09 to 0.16 (Romero et al., 1992c; Grattard et al., 1995; Horowitz et al., 1995).

#### Association of Genital Mycoplasma Presence With Adverse Pregnancy Outcomes

**FIGURE 7:**
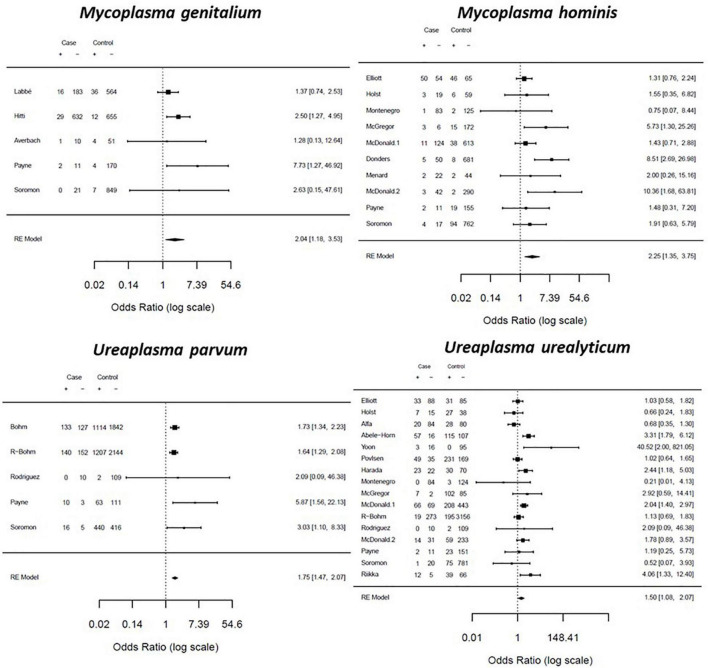
Forest plot for the association between genital Mycoplasma infection and preterm delivery. Effect sizes were presented as pooled odds ratio with 95% confidence interval.

To determine whether genital mycoplasma presence is associated with adverse pregnancy outcomes, we performed a MA for all case-control and cohort studies with reported odds ratio for genital mycoplasma infection and adverse pregnancy outcomes. Women with PTB had significantly higher odds of presence of with all four Mycoplasmas species than women who delivered at term. The highest odds ratio was for *M. hominis* (OR: 2.25; CI: 1.35–3.75; *I*^2^: 44%), followed by *M. genitalium* (OR: 2.04; CIL 1.18–3.53; *I*^2^: 20%), *U. parvum* (OR: 1.75; CI: 1.47–2.07; *I*^2^: 0%), and *U. urealyticum* (OR: 1.50; CI: 1.08–2.07; *I*^2^: 58%) (Figure 7).

**FIGURE 8:**
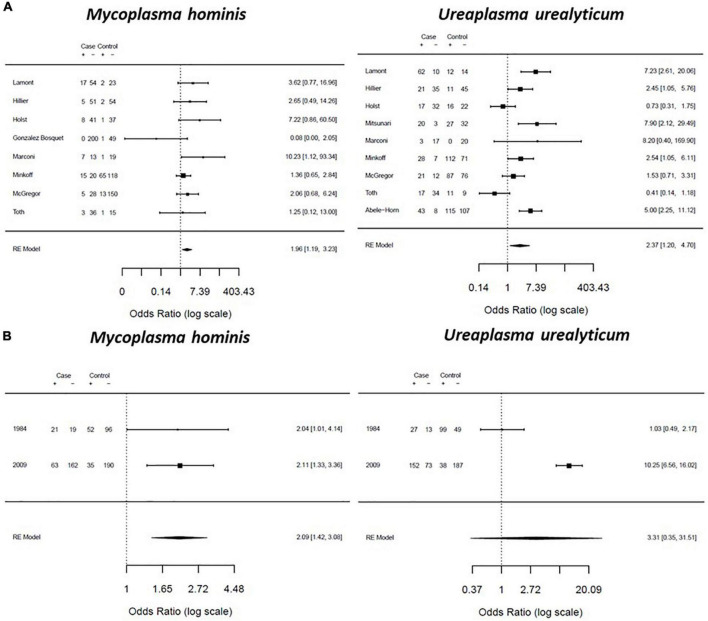
Forest plot for the association between genital Mycoplasma infection and preterm labor **(A)** and preterm prelabor rupture of membranes (PPROM) **(B)**. Effect sizes were presented as pooled odds ratio with 95% confidence interval.

Women who experienced SPTL had significantly higher odds of *M. hominis* (OR: 1.96; CI: 1.19–3.23; *I*^2^: 1%) or *U. urealyticum* (OR: 2.37; CI: 1.20–4.70; *I*^2^: 76%) presence compared to women who did not experience SPTL (Figure 8A). Based on two studies, women with PPROM had significantly higher odds of colonization with *M. hominis* (OR: 2.09; CI: 1.42–3.08; *I*^2^: 0%) than pregnant women without PPROM. These two studies were conducted in the Czech Republic and the USA (Minkoff et al., 1984; Kacerovský et al., 2009). They used cervical or vaginal fluid collected either at admission for rupture of membranes or during the first prenatal visit, and genital mycoplasmas were detected by culture. The result for *U. urealyticum* (OR: 3.31; CI: 0.35–31.51; *I*^2^: 96%) was equivocal and did not reach statistical significance (Figure 8B). No studies reported the odds ratio for *M. genitalium* and *U. parvum* presence in women with SPTL and PPROM.

We performed subgroup analysis on the association of genital mycoplasma with adverse pregnancy outcomes based on the diagnostic method used to detect genital mycoplasmas. The pooled ORs for studies that used culture-based detection methods showed significantly higher odds of PTB and PPROM than studies that used PCR-based detection methods. *M. hominis* and *U. urealyticum* presence were significantly associated with 2.84- and 1.57-times increased odds of PTB, respectively. Only *M. hominis* presence showed significantly increased odds of PPROM, but not *U. urealyticum* (Table 1). We also performed subgroup analysis based on the sample used. We generally observed a higher pooled OR for studies that detected genital mycoplasmas in fetal tissues than in studies that used maternal tissues. For example, the presence of *U. parvum* and *U. urealyticum* in fetal samples had 2.08- and 1.55-times increased odds of PTB. In comparison, *U. parvum* and *U. urealyticum* colonization in the maternal samples was associated with 1.77- and 1.45-times increased odds of PTB. Moreover, *M. hominis* presence of the fetal tissues was associated with 4.35-times increased odds of PTL, whereas *M. hominis* colonization of the maternal tissues was only associated with 1.72-times increased odds of PTL. Lastly, only *M. hominis* and not *U. urealyticum* colonization of the maternal tissues was associated with increased odds of PPROM (2.07-times) (Table 2).

**TABLE 1:** Subgroup analysis of the association (odds ratio) of genital mycoplasma with preterm birth (PTB), preterm labor (PTL), and preterm prelabor rupture of membranes (PPROM) based on the diagnostic method used to detect genital mycoplasma.

Genital mycoplasma	Culture		PCR	
	OR	95% CI	OR	95% CI
**Preterm birth**				
*M. genitalium*	–	–	2.04	1.18–3.52
*M. hominis*	2.84	1.02–2.40	1.64	0.75–3.59
*U. parvum*	–	–	1.75	1.47–2.07
*U. urealyticum*	1.57	1.02–2.40	1.32	0.83–2.12
**Preterm labor**				
*M. genitalium*	–	–	–	–
*M. hominis*	1.78	0.08–1.08	10.23	0.11–4.54
*U. parvum*	–	–	–	–
*U. urealyticum*	1.96	−0.05–1.40	7.95	0.86–3.28
**Preterm prelabor rupture of membrane**				
*M. genitalium*	–	–	–	–
*M. hominis*	2.09	1.42–3.08	–	–
*U. parvum*	–	–	–	–
*U. urealyticum*	3.31	0.35–31.50	–	–

**TABLE 2:** Subgroup analysis of the association (odds ratio) of genital mycoplasma with preterm birth (PTB), preterm labor (PTL), and preterm prelabor rupture of membranes (PPROM) based on the specimen used to detect genital mycoplasma.

Genital mycoplasma	Fetal sample		Maternal sample		Both samples	
	OR	95% CI	OR	95% CI	OR	95% CI
**Preterm birth**						
*M. genitalium*	–	–	2.04	1.18–3.52	–	–
*M. hominis*	0.75	0.07–8.44	2.37	1.39–4.04	–	–
*U. parvum*	2.08	0.09–46.06	1.74	1.47–2.07	–	–
*U. urealyticum*	1.55	0.22–11.12	1.45	1.09–1.94	3.31	1.79–6.12
**Preterm labor**						
*M. genitalium*	–	–	–	–	–	–
*M. hominis*	4.35	1.14–16.59	1.72	1.01–2.91	–	–
*U. parvum*	–	–	–	–	–	–
*U. urealyticum*	2.68	1.18–6.09	1.94	0.77–4.90	5.00	2.25–11.12
**Preterm prelabor rupture of membrane**						
*M. genitalium*	–	–	–	–	–	–
*M. hominis*	–	–	2.09	1.42–3.08	–	–
*U. parvum*	–	–	–	–	–	–
*U. urealyticum*	–	–	3.31	0.35–31.50	–	–

## Discussion

### Main Findings

This SR and MA showed a 28% prevalence of *U. parvum* mycoplasmas among women with PTB, 19% prevalence of *U. urealyticum* in SPTL, and 27% in women with PPROM. We also showed that detection of genital mycoplasmas was significantly associated with a one to two-fold higher risk for PTB and a two to three-fold higher risk for SPTL and PPROM. However, after subgroup analysis, the prevalence and the association between genital mycoplasma and adverse pregnancy outcomes changes depending on the diagnostic test and the sample used. We observed that genital mycoplasmas have a higher prevalence in maternal samples than fetal samples. Genital mycoplasma presence of the cervix and vagina was associated with lower odds of PTB and PTL.

In contrast, colonization of the AF, fetal membrane, and the placenta was associated with higher odds of PTL. In addition, the association (odds ratio) of GM with adverse pregnancy outcomes changed after subgroup analysis (Tables 1, 2). This further adds to the growing evidence that genital mycoplasma presence in the cervix and the vagina is insufficient to promote preterm birth. Unless the cervix is compromised, it will serve as a barrier protecting the fetus in the intraamniotic cavity (Motomura et al., 2020; Pavlidis et al., 2020). Genital mycoplasma in the cervicovaginal space may promote a mild inflammatory response to prime the cervical tissues to mount an immune response to potentially pathogenic organisms that may try to ascend to the amniotic cavity (Tantengco and Menon, 2022).

Ascending genital mycoplasma to the fetal side very well may cause adversity during pregnancy as the presence in the amniotic cavity or invasion of the fetal organs can mount a massive fetal inflammatory response that can cause SPTL and PTB. This is unlikely for infections limited to the maternal side alone. We propose that the fetal inflammatory response is the final trigger to promote genital mycoplasma-induced PTB, while maternal inflammation due to GM presence helps maintain immunologic homeostasis during pregnancy and promote fetoplacental growth.

Our data partly corroborate the results of two recent SRs, which showed that *M. genitalium*, but not *M. hominis*, *U. urealyticum*, or *U. parvum* was significantly associated with PTB (Lis et al., 2015; Ma et al., 2021). Those SRs analyzed only three articles reporting PTB with *M. hominis* or *U. urealyticum* presence and one article regarding *U. parvum*. At the same time, our SR also included more recent papers and retried respectively 10, 16, and 5 articles reporting an association between these microorganisms and PTB. It is also important to highlight that one of the two referred SRs included only studies based on NAATs to detect genital mycoplasmas. In contrast, our SR had studies that used other diagnostic tests such as culture and PCR-based tests.

### Strengths and Limitations

Most of the included studies were from Europe (33/93) or North American (30/93). This limited number of studies from some regions, such as low- and middle-income countries, impaired an estimative of a global prevalence and a more reliable association between the genital mycoplasmas and adverse pregnancy outcomes. Therefore, more studies are required in these regions, especially following a recent SR that showed a higher prevalence of *M. genitalium* infection in the general population in developing countries compared to developed countries (Baumann et al., 2018). Based on cross-sectional studies, the prevalence of *M. hominis* among women with SPTL or PTB was >10% in AF samples from United States (Gravett et al., 1986) and Switzerland (Nguyen et al., 2004), in vaginal samples from United States (Gravett et al., 1986; Harger et al., 1991), Israel (Horowitz et al., 1995), and Korea (Choi et al., 2012), and in fetal membranes from United Kingdom (Cahill et al., 2005). With PPROM, over 10% prevalence of *M. hominis* was reported in vaginal samples from France (Grattard et al., 1995) and Israel (Horowitz et al., 1995), in AF samples from United States (Perni et al., 2004) and Czech Republic (Kacerovsky et al., 2014), and cord blood samples from Czech Republic (Kacerovsky et al., 2014). In contrast, the prevalence of *U. urealyticum* in SPTL and/or PTB was more than 30% in AF samples from United States (Gravett et al., 1986; Athayde et al., 2000; Romero et al., 2019) and Israel (Horowitz et al., 1995; Bashiri et al., 1999), in vaginal samples from United States (Gravett et al., 1986; Harger et al., 1991), Israel (Holst et al., 1994), France (Grattard et al., 1995; Goffinet et al., 2003), Germany (Abele-Horn et al., 1997), Korea (Choi et al., 2012), Japan (Usui et al., 2002), Denmark/England (Thomsen et al., 1984), and Czech Republic (Koucký et al., 2016), while more than 30% of *U. urealyticum* in PPROM was reported in AF samples from United States (Romero et al., 1992a; Font et al., 1995; Athayde et al., 2000; Pacora et al., 2000; Perni et al., 2004), Israel (Horowitz et al., 1995), and Austria (Witt et al., 2005), in vaginal samples in France (Grattard et al., 1995), and placenta in Austria (Witt et al., 2005). These data demonstrate that the most prevalent microorganisms have been reported in diverse populations. This review observed significant associations in only two studies from low-income countries. These data demonstrate that the most prevalent microorganisms have been reported in diverse populations. This review observed significant associations in only two studies from low-income countries.

Analyses of cord blood cultures in the Alabama Preterm Birth Study demonstrated that 27.9% of samples from non-white women and 16.8% from white women were positive for *U. urealyticum* and/or *M. hominis* (Goldenberg et al., 2008). There was also a lower prevalence of *M. hominis* in white women, albeit that *U. urealyticum* presence was unrelated to ethnicity (Witt et al., 2005). Racial or ethnic disparities in adverse pregnancy outcomes associated with the genital mycoplasmas are difficult to establish based on current data as many studies do not report population ethnicity and we strongly recommend inclusion of race/ethnicity of populations in future studies to determine the contribution of racial and genetic factors in the determination of risk.

The relationship between intraamniotic infection and adverse pregnancy outcomes has been examined extensively over the last four decades. In this MA we were able to confirm the prevalence and importance of the genital mycoplasmas in SPTL, PTB and PPROM. The human *Ureaplasma* spp. are divided into *U. urealyticum* and *U. parvum* with 14 known serotypes. In the raw prevalence analysis, ureaplasmas were predominant in the three studied outcomes and was more prevalent in SPTL and PPROM, while *U. parvum* showed a higher prevalence in PTB. Based on *in vitro* models (Novy et al., 2009; Motomura et al., 2020) and *in vivo* (Shimizu et al., 2008; Novy et al., 2009; Dando et al., 2012; Uchida et al., 2013), it has been argued that injection of *Ureaplasma* and or its antigens may elicit a fetal inflammatory response contributing to PTB, which supports our findings.

Polymicrobial presence demonstrated in PTB (Thomsen et al., 1984; Elliott et al., 1990; McGregor et al., 1990; Hillier et al., 1991; McDonald et al., 1992, 1994; Holst et al., 1994; Grattard et al., 1995; Kundsin et al., 1996; Usui et al., 2002; Goffinet et al., 2003; Goldenberg et al., 2008; Onderdonk et al., 2008; Montenegro et al., 2019), SPTL (Minkoff et al., 1984; Gravett et al., 1986; Lamont et al., 1987; Hillier et al., 1988; Romero et al., 1989, 1992c; McGregor et al., 1990; Harger et al., 1991; Toth et al., 1992; Watts et al., 1992; Holst et al., 1994, 2005; Font et al., 1995; Horowitz et al., 1995; Marconi et al., 2011; Choi et al., 2012; Kim et al., 2012; Lee et al., 2013), and PPROM (Minkoff et al., 1984; Romero et al., 1992b,c; Averbuch et al., 1995; Font et al., 1995; Grattard et al., 1995; Horowitz et al., 1995; Kundsin et al., 1996; Perni et al., 2004; Shim et al., 2005; Nasution et al., 2007), was mostly based on a combination of *U. urealyticum* and *M. hominis* (Ma et al., 2021). Co-infections were also associated with decreased gestational age at birth and birth weight, and a significant increase in the incidence of histologic chorioamnionitis (Kwak et al., 2014). However, most of these studies examined only genital mycoplasmas. They did not examine other more virulent Gram-negative or positive associated with mycoplasmas, which may have biased some reports and conclusions.

One of the limitations of this SR is the heterogeneity shown by the large *I*^2^ values from our MA. This may be due to several factors, such as the variability in the source and timing of biological samples and diagnostic methods for detecting genital mycoplasma.

### Interpretation

Like other organisms associated with SPTL, PTB, or PPROM such as *G. vaginalis*, quantitative analysis of the genital mycoplasmas may be more important than simply the presence or absence of the organism, whether detected by cultivation-dependent or independent techniques (Lamont et al., 2020). Using cultivation-dependent technology in women delivering between 26 and 34 completed weeks of gestation, 80 and 24% of those in SPTL were colonized by *U. urealyticum* and *M*. *hominis*, respectively. In contrast, women who were not in SPTL but delivered electively for maternal-fetal indications such as preeclampsia or intrauterine growth restriction at the same gestational age, 46 and 8%, were colonized by *U. urealyticum* and *M. hominis*, respectively. The qualitative analysis was statistically significantly different for *U. urealyticum* (*p* < 0.01) but not for *M. hominis.* Nevertheless, when only heavy presence of *M. hominis* was considered [>10^5^ color change units (the unit of quantity at the time)], 13 women in the study group (18%) and none of the controls were identified (*p* < 0.05), suggesting that it was not the presence or absence of the organism that was important but the quantity present (Lamont et al., 1987). This was also supported by various *in vitro* and *in vivo* studies showing a dose-dependent increase in inflammation and PTB rate with higher doses of *Ureaplasma* spp. infections (Glaser et al., 2017, 2018; Motomura et al., 2020; Tantengco et al., 2021).

Only specific subtypes of the genital mycoplasmas are pathogenic. Using molecular-based techniques, it was found that *Ureaplasma* species consist of 14 serovars from two biovars. The majority of human *Ureaplasma* isolates belong to *U. parvum* (biovar 1), comprising four serovars (the predominant biovar in patients with genital tract infections), with *U. urealyticum* (biovar 2) containing ten serovars isolated much less often. However, the data are limited and conflicted because of the difficulties with traditional genotyping methods (Dhawan et al., 2012). This may be relevant to the confusion about the role of ureaplasmas in adverse outcomes of pregnancy.

The most common method of identifying microbes reported was culture, followed by PCR. Both these approaches are used for both qualitative and quantitative detection of bacteria. Most recent studies used molecular detection of genital mycoplasma instead of culture, as only four studies used microbiological culture after 2010 (Kim et al., 2012; Lee et al., 2013; Wang et al., 2013; Suzuki et al., 2018). Accordingly, detection of genital mycoplasmas may have been underestimated in former studies as the detection rate of mycoplasmas by PCR is higher than that by cultivation (Choi et al., 2012). Molecular analysis has the advantage of differentiating mycoplasma species and allowing the bacterial load to be measured, although bacterial load was reported only in a few included studies. The microbial load can alter the type of biomarker and their quantities (Flores-Herrera et al., 2012; Noda-Nicolau et al., 2016). Sample collections for mycoplasma detection were mainly performed at admission with clinical indications of labor and/or at the time of labor and delivery.

One of the objectives of this study was to determine the association between the genital mycoplasmas and adverse pregnancy outcomes with specific inflammatory biomarkers. However, the data were too heterogeneous to perform a MA. It is still unclear if the genital mycoplasmas can mount a proinflammatory surge that can lead to adverse pregnancy outcomes. Most biomarker studies examined only proinflammatory markers, and few studies report anti-inflammatory mediators. Among PPROM patients, Oh et al. (2010) have demonstrated higher maternal blood WBC and CRP during mycoplasma AF positivity, mainly *Ureaplasma* spp. (Oh et al., 2010).

Conversely, one major study examined 26 proteins in AF and reported several anti-inflammatory markers (IL-10, sTNFR1, sIL-6r) elevated in cases associated with the genital mycoplasmas, also predominantly by Ureaplasmas (Cobo et al., 2012). *In vitro* models have suggested that unbiased analysis of pro- and anti-inflammatory markers is required to determine the actual role of diverse mycoplasmas in inducing an inflammatory response during pregnancy (Noda-Nicolau et al., 2016; Polettini et al., 2018). Some genital mycoplasmas such as *Ureaplasma* spp. may present themselves as proxies for other virulent microbial pathogens such as *E. coli*, Group B streptococcus (GBS), or *Gardnerella* species. Therefore, genital mycoplasma might be insufficient to cause SPTL and PTB but can assist other pathogens or modify the tissue environment in a polymicrobial situation.

## Conclusion

This SR raises several questions related to the pathogenic role of the genital mycoplasmas, and the MA found an association with specific adverse pregnancy outcomes between a single pathogen or in mycoplasmas combination. However, ambiguity remains concerning independent contributions by various genital mycoplasmas and adverse pregnancy outcomes. This ambiguity is derived from a lack of evidence on the precise location of the infection (fetal vs. maternal), microbial load (most reports on load are based on amplified microbial nucleic acid), and detection timing. This SR also evaluated biomarkers, and we report that the genital mycoplasmas are poor immunogens and do not generate the quantity of inflammatory mediators like those of other pathogens. However, this conclusion may depend on their load, location and polymicrobial presence. Our own *in vitro* studies have shown that poor immunogenicity by these microbes in human fetal membranes as well as in the cervix (Noda-Nicolau et al., 2016; Tantengco et al., 2021, 2022). These results suggest that the association between the genital mycoplasmas and adverse pregnancy outcomes might not be strong enough to warrant universal antimicrobial intervention. Intervention to prevent PTB may not be recommended but based on several factors such as the infectious agent, polymicrobial etiology, knowledge of the most virulent pathogen, bacterial load and location, and subsequent neonatal morbidity intervention strategies may be indicated and tailored to the patient’s needs.

## Author Contributions

RM: conception of the work and funding. NN-N and MGS: data collection. NN-N, MGS, JP, CM, MCS, GB, GC, and OT: data extraction. NN-N, JP, OT, MCS, GB, and GC: quality assessment and drafting the manuscript. JP: meta-analysis. RM, RL, and BT: critical revision of the manuscript. All authors reviewed and approved the final version of the manuscript.

## Conflict of Interest

The authors declare that the research was conducted in the absence of any commercial or financial relationships that could be construed as a potential conflict of interest.

## Publisher’s Note

All claims expressed in this article are solely those of the authors and do not necessarily represent those of their affiliated organizations, or those of the publisher, the editors and the reviewers. Any product that may be evaluated in this article, or claim that may be made by its manufacturer, is not guaranteed or endorsed by the publisher.
