# Supplementary material for: Genital Mycoplasmas and Biomarkers of Inflammation and Their Association With Spontaneous Preterm Birth and Preterm Prelabor Rupture of Membranes: A Systematic Review and Meta-Analysis
Source: Front Microbiol. 2022 Mar 30;13:859732. doi: 10.3389/fmicb.2022.859732 (PMC9006060; doi:10.3389/fmicb.2022.859732)
Supplement: Supplementary file 1 [file Data_Sheet_1.pdf]

## Supplementary Figures

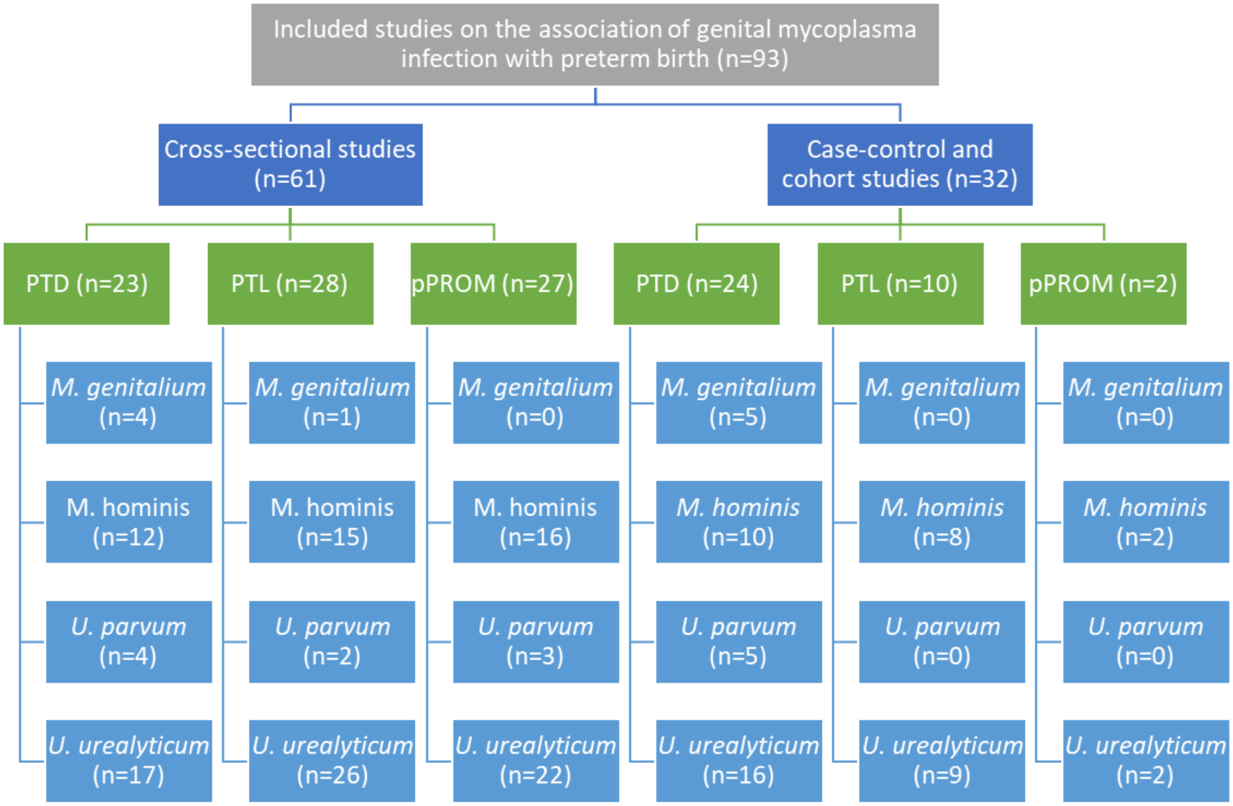


**Supplementary Figure 1.** Summary diagram showing the distribution of studies on the association of different species (*M. genitalium*, *M. hominis*, *U. parvum*, and *U. urealyticum*) causing genital Mycoplasma infection with pregnancy outcomes (SPTL, PTB, and PPROM)
