# Supplementary material for: Genital Mycoplasmas and Biomarkers of Inflammation and Their Association With Spontaneous Preterm Birth and Preterm Prelabor Rupture of Membranes: A Systematic Review and Meta-Analysis
Source: Front Microbiol. 2022 Mar 30;13:859732. doi: 10.3389/fmicb.2022.859732 (PMC9006060; doi:10.3389/fmicb.2022.859732)
Supplement: Supplementary file 2 [file Table_1.docx]

**Supplementary Table 1.** Quality Assessment (Cross-Sectional Studies)

| **Title** | **Author** | **Selection** | | | | **Comparability** | **Outcomes** | | **Total** |
| --- | --- | --- | --- | --- | --- | --- | --- | --- | --- |
|  |  | Representativeness of the sample | Sample size | Non-respondents | Ascertainment of the exposure (genital Mycoplasma infection) | The subjects in different outcome groups are comparable, based on the study design or analysis. Confounding factors are controlled | Assessment of the outcome | Statistical test |  |
| Abele | 1997 | 0 | 1 | 1 | 1 | 0 | 2 | 1 | 6 |
| Allen-Daniels | 2015 | 0 | 0 | 1 | 1 | 1 | 2 | 1 | 6 |
| Athayde | 2000 | 1 | 0 | 1 | 1 | 0 | 2 | 0 | 5 |
| Athayde | 2000 | 1 | 0 | 0 | 1 | 1 | 2 | 1 | 6 |
| Averbuch | 1995 | 1 | 1 | 1 | 1 | 2 | 2 | 1 | 9 |
| Bashiri | 1999 | 1 | 1 | 1 | 2 | 0 | 2 | 0 | 7 |
| Bretelle | 2014 | 0 | 1 | 1 | 2 | 1 | 2 | 1 | 8 |
| Cahill | 2005 | 1 | 1 | 1 | 2 | 0 | 2 | 1 | 8 |
| Carroll | 1995 | 1 | 1 | 1 | 1 | 1 | 2 | 1 | 8 |
| Cherouny | 1993 | 0 | 0 | 0 | 1 | 1 | 2 | 1 | 5 |
| Choi | 2012 | 1 | 1 | 1 | 2 | 0 | 2 | 1 | 8 |
| Cox | 2016 | 1 | 0 | 1 | 2 | 0 | 0 | 1 | 5 |
| Espinoza | 2002 | 1 | 0 | 1 | 1 | 0 | 2 | 1 | 6 |
| Figueroa | 2005 | 0 | 1 | 1 | 1 | 1 | 2 | 1 | 7 |
| Font | 1995 | 1 | 0 | 1 | 1 | 0 | 2 | 1 | 6 |
| Foulon | 1995 | 1 | 0 | 1 | 2 | 0 | 2 | 0 | 6 |
| Gardella | 2004 | 1 | 0 | 1 | 2 | 0 | 2 | 1 | 7 |
| Gauthier | 1994 | 1 | 1 | 1 | 1 | 1 | 2 | 1 | 8 |
| Goffinet | 2003 | 1 | 1 | 1 | 1 | 2 | 2 | 1 | 9 |
| Goldenberg | 2008 | 1 | 1 | 1 | 2 | 1 | 2 | 1 | 9 |
| Grattard | 1995 | 0 | 1 | 1 | 1 | 0 | 2 | 1 | 6 |
| Gravett | 1986 | 1 | 0 | 1 | 2 | 0 | 2 | 1 | 7 |
| Harger | 1991 | 1 | 0 | 0 | 2 | 0 | 2 | 1 | 6 |
| Hazan | 1995 | 1 | 0 | 1 | 2 | 0 | 2 | 1 | 7 |
| Hillier | 1991 | 1 | 1 | 0 | 2 | 1 | 2 | 1 | 8 |
| Holst | 2005 | 1 | 0 | 1 | 1 | 0 | 2 | 1 | 6 |
| Horowitz | 1995 | 1 | 0 | 1 | 2 | 0 | 2 | 1 | 7 |
| Horowitz | 1995 | 1 | 0 | 1 | 2 | 0 | 0 | 0 | 4 |
| Jacobsson | 2003 | 1 | 0 | 1 | 2 | 0 | 2 | 1 | 7 |
| Jacobsson | 2005 | 1 | 0 | 1 | 2 | 0 | 2 | 1 | 7 |
| Jacobsson | 2003 | 1 | 1 | 1 | 1 | 2 | 2 | 1 | 9 |
| Jacobsson | 2003 | 1 | 0 | 1 | 2 | 0 | 2 | 1 | 7 |
| Jacobsson | 2009 | 1 | 0 | 0 | 2 | 0 | 0 | 1 | 4 |
| Jayaprakash | 2016 | 1 | 1 | 1 | 1 | 2 | 2 | 1 | 9 |
| Kacerovsky | 2009 | 1 | 1 | 1 | 1 | 1 | 2 | 1 | 8 |
| Kacerovsky | 2014 | 1 | 1 | 1 | 1 | 2 | 2 | 1 | 9 |
| Kasper | 2010 | 1 | 1 | 1 | 1 | 1 | 2 | 1 | 8 |
| Kim | 2012 | 1 | 0 | 1 | 2 | 2 | 2 | 1 | 9 |
| Kim | 2003 | 0 | 0 | 1 | 1 | 1 | 2 | 1 | 6 |
| Koucký | 2016 | 1 | 0 | 1 | 2 | 0 | 2 | 1 | 7 |
| Kundsin | 1996 | 1 | 1 | 1 | 1 | 2 | 2 | 1 | 9 |
| Lee | 2013 | 1 | 0 | 1 | 2 | 2 | 2 | 1 | 9 |
| Leli | 2018 | 1 | 1 | 1 | 2 | 0 | 2 | 1 | 8 |
| Lu | 2001 | 1 | 1 | 1 | 1 | 2 | 2 | 1 | 9 |
| Musilova | 2017 | 1 | 1 | 1 | 1 | 2 | 2 | 1 | 9 |
| Musilova | 2017 | 1 | 1 | 1 | 1 | 2 | 2 | 1 | 9 |
| Nasution | 2007 | 1 | 1 | 1 | 1 | 1 | 2 | 1 | 8 |
| Nguyen | 2004 | 1 | 1 | 1 | 2 | 0 | 2 | 1 | 8 |
| Oh | 2010 | 1 | 1 | 1 | 1 | 2 | 2 | 1 | 9 |
| Oh | 2019 | 1 | 0 | 0 | 2 | 0 | 2 | 1 | 6 |
| Olomu | 2009 | 1 | 1 | 1 | 1 | 2 | 2 | 1 | 9 |
| Onderdonk | 2008 | 1 | 1 | 1 | 2 | 0 | 2 | 0 | 7 |
| Pacora | 2000 | 1 | 1 | 0 | 2 | 0 | 2 | 1 | 7 |
| Park | 2013 | 1 | 1 | 0 | 2 | 0 | 2 | 1 | 7 |
| Paul | 1998 | 1 | 0 | 1 | 1 | 0 | 2 | 1 | 6 |
| Perni | 2004 | 1 | 1 | 1 | 1 | 2 | 2 | 1 | 9 |
| Romero | 1992 | 1 | 0 | 1 | 2 | 0 | 2 | 1 | 7 |
| Romero | 1989 | 1 | 1 | 1 | 2 | 0 | 2 | 1 | 8 |
| Romero | 1992 | 1 | 1 | 1 | 2 | 0 | 2 | 1 | 8 |
| Romero | 2019 | 1 | 0 | 1 | 1 | 1 | 2 | 1 | 7 |
| Romero | 2014 | 1 | 1 | 1 | 1 | 2 | 2 | 1 | 9 |
| Shim | 2005 | 1 | 1 | 1 | 1 | 2 | 2 | 1 | 9 |
| Suzuki | 2018 | 1 | 1 | 1 | 1 | 2 | 2 | 1 | 9 |
| Sweeney | 2016 | 1 | 1 | 1 | 2 | 0 | 2 | 1 | 8 |
| Thomsen | 1984 | 0 | 1 | 1 | 1 | 0 | 0 | 0 | 3 |
| Usui | 2002 | 1 | 1 | 1 | 1 | 2 | 2 | 1 | 9 |
| Wang | 2013 | 1 | 1 | 1 | 1 | 0 | 2 | 1 | 7 |
| Watts | 1992 | 1 | 0 | 0 | 2 | 0 | 2 | 1 | 6 |
| Watts | 1992 | 1 | 0 | 0 | 1 | 1 | 2 | 1 | 6 |
| Witt | 2005 | 1 | 0 | 1 | 2 | 0 | 2 | 1 | 7 |
| Yoneda | 2017 | 1 | 0 | 1 | 2 | 0 | 2 | 1 | 7 |
| Yoon | 1998 | 1 | 1 | 1 | 2 | 1 | 2 | 1 | 9 |
| Yoon | 2003 | 1 | 1 | 1 | 2 | 0 | 2 | 1 | 8 |
| Yoon | 1998 | 1 | 1 | 1 | 2 | 1 | 2 | 1 | 9 |
| Yoon | 2001 | 1 | 1 | 1 | 2 | 0 | 0 | 1 | 6 |
| Yoon | 1998 | 0 | 0 | 1 | 0 | 1 | 0 | 1 | 3 |

**Assessment:**

Very good: 9 - 10

Good: 7 - 8

Satisfactory: 5 - 6

Unsatisfactory: 0 - 4
