# Supplementary material for: Genital Mycoplasmas and Biomarkers of Inflammation and Their Association With Spontaneous Preterm Birth and Preterm Prelabor Rupture of Membranes: A Systematic Review and Meta-Analysis
Source: Front Microbiol. 2022 Mar 30;13:859732. doi: 10.3389/fmicb.2022.859732 (PMC9006060; doi:10.3389/fmicb.2022.859732)
Supplement: Supplementary file 3 [file Table_2.docx]

**Supplementary Table 2**. Quality Assessment (Case-Control Studies)

| **Author** | **Title** | **Selection** | | | | **Comparability** | **Outcomes** | | | **Total** |
| --- | --- | --- | --- | --- | --- | --- | --- | --- | --- | --- |
|  |  | Is the case definition adequate? | Representativeness of the cases | Selection of Controls | Definition of Controls | Comparability of the cases and controls on the basis of the design or analysis | Ascertainment of exposure | Same method of ascertainment for cases and controls | Non-Response rate |  |
| Abele-Horn | 2009 | 1 | 1 | 0 | 1 | 1 | 1 | 1 | 1 | 7 |
| Alfa | 1995 | 1 | 1 | 0 | 1 | 1 | 1 | 1 | 1 | 7 |
| Daskalakis | 2009 | 1 | 1 | 0 | 1 | 1 | 1 | 1 | 1 | 7 |
| Elliott | 1990 | 1 | 1 | 0 | 1 | 1 | 1 | 1 | 1 | 7 |
| Gonzalez Bosquet | 2006 | 1 | 0 | 1 | 1 | 1 | 1 | 1 | 1 | 7 |
| Harada | 2008 | 0 | 1 | 1 | 1 | 1 | 1 | 1 | 0 | 6 |
| Hillier | 1988 | 1 | 1 | 0 | 1 | 1 | 1 | 1 | 1 | 7 |
| Hitti | 2010 | 1 | 1 | 1 | 1 | 2 | 1 | 1 | 1 | 9 |
| Holst | 1994 | 1 | 1 | 0 | 1 | 1 | 1 | 1 | 1 | 7 |
| Kacerovsky | 2009 | 0 | 0 | 1 | 0 | 1 | 1 | 1 | 1 | 5 |
| Labbé | 2002 | 0 | 1 | 1 | 0 | 1 | 1 | 1 | 0 | 5 |
| Lamont | 1987 | 0 | 0 | 1 | 0 | 1 | 1 | 1 | 1 | 5 |
| Marconi | 2011 | 0 | 1 | 1 | 1 | 0 | 1 | 1 | 0 | 5 |
| Mitsunari | 2005 | 1 | 1 | 1 | 0 | 1 | 1 | 1 | 1 | 7 |
| Montenegro | 2019 | 1 | 1 | 1 | 0 | 1 | 1 | 1 | 1 | 7 |
| Povlsen | 2001 | 1 | 1 | 0 | 1 | 1 | 1 | 1 | 1 | 7 |
| Riikka | 2002 | 0 | 0 | 1 | 0 | 1 | 0 | 1 | 1 | 4 |
| Thomakos | 2009 | 1 | 1 | 0 | 1 | 1 | 1 | 1 | 1 | 7 |
| Yoon | 2001 | 1 | 1 | 0 | 1 | 1 | 1 | 1 | 1 | 7 |

**Assessment:**

Very good: 8 - 9

Good: 6 - 7

Satisfactory: 5

Unsatisfactory: 0 - 4
