# Supplementary material for: Genital Mycoplasmas and Biomarkers of Inflammation and Their Association With Spontaneous Preterm Birth and Preterm Prelabor Rupture of Membranes: A Systematic Review and Meta-Analysis
Source: Front Microbiol. 2022 Mar 30;13:859732. doi: 10.3389/fmicb.2022.859732 (PMC9006060; doi:10.3389/fmicb.2022.859732)
Supplement: Supplementary file 4 [file Table_3.docx]

**Supplementary Table 3.** Quality Assessment (Cohort Studies)

| **Author** | **Year** | **Selection** | | | | **Comparability** | **Outcomes** | | | **Total Score** |
| --- | --- | --- | --- | --- | --- | --- | --- | --- | --- | --- |
|  |  | Representativeness of the exposed cohort | Selection of the non-exposed cohort | Ascertainment of exposure | Demonstration that outcome of interest was not present at start of study | Comparability of cohorts on the basis of the design or analysis | Assessment of outcome | Was follow-up long enough for outcomes to occur | Adequacy of follow up of cohorts |  |
| Averbach | 2013 | 1 | 1 | 1 | 1 | 2 | 1 | 1 | 1 | 9 |
| Barton | 2003 | 1 | 1 | 1 | 1 | 0 | 0 | 1 | 1 | 6 |
| Bohm | 2019 | 1 | 1 | 1 | 1 | 0 | 1 | 1 | 1 | 7 |
| Cobo | 2017 | 1 | 1 | 1 | 1 | 2 | 0 | 1 | 0 | 7 |
| Donders | 2009 | 1 | 1 | 1 | 1 | 0 | 1 | 1 | 1 | 7 |
| Edwards | 2006 | 1 | 1 | 1 | 1 | 2 | 1 | 1 | 1 | 9 |
| Kacerovsky | 2014 | 1 | 1 | 1 | 1 | 0 | 1 | 1 | 1 | 7 |
| Kacerovsky | 2013 | 1 | 1 | 1 | 1 | 0 | 1 | 1 | 1 | 7 |
| Kacerovsky | 2012 | 1 | 1 | 1 | 1 | 0 | 1 | 1 | 1 | 7 |
| Kacerovsky | 2014 | 1 | 1 | 1 | 1 | 2 | 0 | 1 | 1 | 8 |
| Kacerovsky | 2015 | 0 | 1 | 1 | 1 | 2 | 1 | 1 | 1 | 8 |
| McDonald | 1992 | 1 | 1 | 1 | 1 | 2 | 1 | 1 | 1 | 9 |
| McDonald | 1994 | 1 | 1 | 1 | 1 | 0 | 1 | 1 | 1 | 7 |
| McGregor | 1990 | 1 | 1 | 1 | 1 | 0 | 1 | 1 | 0 | 6 |
| Menard | 2010 | 1 | 1 | 1 | 1 | 0 | 1 | 1 | 1 | 7 |
| Minkoff | 1984 | 1 | 1 | 1 | 1 | 0 | 1 | 1 | 1 | 7 |
| Musilova | 2017 | 1 | 1 | 1 | 1 | 2 | 0 | 1 | 1 | 8 |
| Payne | 2016 | 1 | 1 | 1 | 1 | 0 | 1 | 1 | 1 | 7 |
| Payne | 2014 | 1 | 1 | 1 | 1 | 2 | 1 | 1 | 1 | 9 |
| Rittenschober-Bohm | 2018 | 1 | 1 | 1 | 1 | 0 | 1 | 1 | 1 | 7 |
| Rodriguez | 2011 | 1 | 1 | 1 | 1 | 0 | 1 | 1 | 0 | 6 |
| Rodriguez-trujillo | 2016 | 1 | 1 | 1 | 1 | 2 | 1 | 1 | 1 | 9 |
| Soromon | 2006 | 1 | 1 | 1 | 1 | 0 | 1 | 1 | 0 | 6 |
| Stepan | 2016 | 1 | 1 | 1 | 1 | 2 | 0 | 1 | 1 | 8 |
| Toth | 1992 | 1 | 1 | 1 | 1 | 0 | 1 | 1 | 0 | 6 |
| Veleminsky | 2008 | 1 | 1 | 1 | 1 | 0 | 0 | 1 | 1 | 6 |
| Vogel | 2006 | 1 | 1 | 1 | 1 | 2 | 1 | 1 | 1 | 9 |
| Wood | 2019 | 1 | 1 | 1 | 1 | 2 | 0 | 1 | 1 | 8 |

**Assessment:**

Very good: 8 - 9

Good: 6 - 7

Satisfactory: 5

Unsatisfactory: 0 - 4
