# Supplementary material for: Genital Mycoplasmas and Biomarkers of Inflammation and Their Association With Spontaneous Preterm Birth and Preterm Prelabor Rupture of Membranes: A Systematic Review and Meta-Analysis
Source: Front Microbiol. 2022 Mar 30;13:859732. doi: 10.3389/fmicb.2022.859732 (PMC9006060; doi:10.3389/fmicb.2022.859732)
Supplement: Supplementary file 6 [file Table_5.docx]

**Supplementary Table 5.** Qualitative synthesis of cross-sectional studies showing the prevalence of genital Mycoplasma infections in SPTL, PTB, and PPROM patients.

| **First author** | **Year** | **Country** | **Time of screening** | **Definition of outcome (PTB, SPTL, pPROM)** | **Specimen Used** | **Method of Detection** | **GA measurement** | ***Mycoplasma genitalium*** | | ***Mycoplasma hominis*** | | ***Ureaplasma parvum*** | | ***Ureaplasma urealyticum*** | |
| --- | --- | --- | --- | --- | --- | --- | --- | --- | --- | --- | --- | --- | --- | --- | --- |
|  |  |  |  |  |  |  |  | **Prevalence (Raw Count)** | **Prevalence** | **Prevalence (Raw Count)** | **Prevalence** | **Prevalence (Raw Count)** | **Prevalence** | **Prevalence (Raw Count)** | **Prevalence** |
| **Preterm birth** | | | | | | | | | | | | | | | |
| Thomsen | 1984 | Denmark, England | 16th weeks of gestation or during c-section/ 2nd or 3rd trimester | NM | C, AF | culture | NM |  |  | 28/297 | 0.0943 |  |  | 148/297 | 0.49832 |
| Hillier | 1991 | USA | Delivery | NM | P | culture | Date of the mother's LMP, fundal height, ultrasonography, and an evaluation of the newborn with a standardized Ballard examination |  |  | 2/112 | 0.0179 |  |  | 24/112 | 0.21429 |
| Romero | 1992 | USA | Midtrimester | NM | AF | culture | NM |  |  |  |  |  |  | 4/15 | 0.26667 |
| Grattard | 1995 | France | After delivery (1 to 3 days) | PTB: ≤37 complete WG | CV | culture and PCR | NM |  |  | 3/8 | 0.3750 |  |  | 4/8 | 0.50000 |
| Abele | 1997 | Germany | After admission | NM | VS | culture and PCR | NM |  |  |  |  |  |  | 72/174 | 0.41379 |
| Paul | 1998 | India | 3rd trimester | NM | VF | culture | NM |  |  |  |  |  |  | 24/303 | 0.07921 |
| Yoon | 1998 | Korea | NM | PTB: ≤36 weeks | AF | culture | NM |  |  |  |  |  |  | 25/120 | 0.20833 |
| Bashiri | 1999 | Israel | 2nd trimester | NM | AF | culture | NM |  |  |  |  |  |  | 1/3 | 0.33333 |
| Pacora | 2000 | Korea | Women at term: before cesarean delivery. In labor or with PROM: uncertain GA | NM | AF | culture | NM |  |  |  |  |  |  | 11/105 | 0.10476 |
| Nguyen | 2004 | Switzerland | 2nd trimester | SPTB: delivery prior to 37 weeks gestation preceded by SPTL | AF | PCR | NM |  |  | 3/28 | 0.1071 |  |  |  |  |
| Onderdonk | 2008 | USA | After delivery (2nd and 3rd trimester) | NM | P | culture and PCR | NM |  |  | 88/1365 | 0.0645 |  |  | 69/1365 | 0.05055 |
| Goldenberg | 2008 | USA | 2nd and 3rd trimester | SPTB: delivery after either spontaneous SPTL or spontaneous PPROM | CB | culture | NM |  |  | 21/351) | 0.0598 |  |  | 43/351 | 0.12251 |
| Wang | 2013 | USA | CB: after delivery |  | AF | culture | LMP and/or ultrasound evaluation | 1/36 (PTB) | 0.02778 |  |  | 5/36 | 0.138889 |  |  |
| Bretelle | 2014 | France | NM | NM | VF | PCR | NM |  |  | 2/220 | 0.0091 |  |  |  |  |
| Sweeney | 2016 | USA | After delivery | Delivery <37 weeks of gestation | P | culture and PCR | NM |  |  |  |  | 27/44 | 0.61364 | 6/44 | 0.13636 |
| Leli | 2018 | Italy | NM | NM | C | PCR | NM | 0/32 | 0.00000 | 1/32 | 0.0313 | 11/32 | 0.34375 | 1/32 | 0.03125 |
| Suzuki | 2018 | Japan | Delivery | NM | V | culture | NM |  |  | 1/54 | 0.0185 |  |  |  |  |
| Romero | 2019 | USA | AF: delivery | NM | AF, VS | culture, PCR, mass spectrometry | Date of the LMP and confirmed by ultrasound examination |  |  |  |  |  |  | 3/6 | 0.50000 |
| Lu | 2001 | USA | Between 21- and 25-weeks’ gestational age | PTB: Birth at <37 weeks’ gestation | VF | PCR | NM | 5/124 | 0.04032 |  |  |  |  |  |  |
| Usui | 2002 | Japan | 6 - 22 weeks of gestation or at the first visit in women who presented with gestation of > 22 weeks. | PTB at < 33 weeks of gestation and at < 37 weeks of gestation | VF | culture | GA and estimated date of delivery were confirmed by ultrasonography. GA at delivery was obtained from the medical record of each woman |  |  | 62/1958 | 0.0317 |  |  | 1031/1958 | 0.52656 |
| Goffinet | 2003 | France | Between 24 and 34 completed weeks’ gestation | Delivery before 33 and 35 complete weeks’ gestation | VF | culture | GA confirmed by a first trimester ultrasonography. |  |  | 1/47 | 0.0213 |  |  | 16/47 | 0.34043 |
| Kundsin | 1996 | USA | At delivery | NM | P | culture | Fetal ultrasound scan estimate obtained before the 13th week of gestation, prenatal record, and, when no other information was available, the GA as recorded in the log of the neonatal intensive care unit (NICU). |  |  | 5/174 | 0.0287 |  |  | 38/174 | 0.21839 |
| **Spontaneous Preterm Labor** | | | | | | | | | | | | | | | |
| Gravett | 1986 | USA | 3rd trimester | SPTL: presence of regular, painful contractions occurring at least every 5 minutes and evidence of cervical dilatation of effacement | AF | culture | NM |  |  | 1/54 | 0.0185 |  |  | 6/54 | 0.11111 |
| Harger | 1991 | USA | 3rd trimester | SPTL: uncomfortable uterine contractions associated with cervical dilatation and effacement, or by at least six uncomfortable uterine contractions within 1 hour | CF | culture | LMP and confirmed by early pregnancy test, uterine size, date of quickening, and serial ultrasound |  |  | 11/35 | 0.3143 |  |  | 17/32 | 0.53125 |
| Watts | 1992 | USA | 2nd and 3rd trimester | SPTL: regular contractions occurring at least every 10 minutes or six times per hour, together with either documented cervical change or a Bishop score of at least 4 that included cervical dilatation of at least 1cm or cervical effacement of at least 50% on the initial examination | AF | culture | Ultrasound examination |  |  | 0/105 | 0.0000 |  |  | 4/105 | 0.03810 |
| Romero | 1992 | USA | NM | SPTL: presence of regular uterine contractions with a frequency of at least two every 10 minutes and with changes in the cervical Bishop's score occurring before 37 completed weeks. | AF | culture | NM |  |  | 0/52 | 0.0000 |  |  | 2/52 | 0.03846 |
| Horowitz | 1995 | Israel | Within 24 hours of PPROM or SPTL | SPTL: presence of regular uterine contractions associated with progressive effacement and dilatation of the uterine cervix. | VF | culture e ELISA | NM |  |  |  |  |  |  | 6/161 | 0.03727 |
| Font | 1995 | USA | Within 24 hours of admission | SPTL: regular uterine contractions associated with cervical change or more than 2cm dilation or 80% effacement on initial cervical examination. | AF | culture | NM |  |  | 0/37 | 0.0000 |  |  | 6/37 | 0.16216 |
| Foulon | 1995 | Belgium | Within 24 hours of admission | SPTL: regular contractions of at least 1 in 10 minutes and a cervical dilation of less than 3 cm | CS, AF | culture | NM |  |  |  |  |  |  | 2/44 | 0.04545 |
| Hazan | 1995 | Israel | NM | SPTL: presence of regular uterine contractions with frequency of 2-3 every 10 minutes associated with changes in cervical effacement (>50%) and dilatation (>1cm). | AF | culture | NM |  |  |  |  |  |  | 3/55 | 0.05455 |
| Horowitz | 1995 | Israel | Mid-trimester | NM | CF | culture | NM |  |  | 9/47 | 0.1915 |  |  | 36/47 | 0.76596 |
| Athayde | 2000 | USA | NM | SPTL: presence of regular uterine contractions occurring with a minimum frequency of 2 every 10 minutes accompanied by changes in either cervical effacement or dilation or both at <37 completed weeks’ gestation. | AF | culture | NM |  |  |  |  |  |  | 14/42 | 0.33333 |
| Espinoza | 2002 | USA | NM | SPTL: regular uterine contractions with a frequency of at least two every 10 min and/or changes in the cervical Bishop’s score occurring before 37 weeks | AF | culture | NM |  |  |  |  |  |  | 4/44 | 0.09091 |
| Jacobsson | 2003 | Sweden | Within 12h after admittance | SPTL: regular uterine contractions (at least two uterine contractions/10min during 30min) in combination with cervical changes | AF | PCR | Routine ultrasound in the second trimester and by the date of their LMP (for 2 samples) |  |  |  |  |  |  | 2/61 | 0.03279 |
| Gardella | 2004 | USA | NM | NM | AF | culture, PCR, and sequencing | NM |  |  |  |  |  |  | 2/132 | 0.01515 |
| Witt | 2005 | Austria | During the course of surgical procedure | NM | AF, P | culture | NM |  |  |  |  |  |  | 10/39 | 0.25641 |
| Jacobsson | 2005 | Sweden | AF: in SPTL; Placenta: after delivery | SPTL: regular uterine contractions (at least two uterine contractions/10 minutes during 30 minutes) in combination with cervical changes: ≤2 cm length+ ≥ 1 cm dilatation; ≤ 2 cm length + cervical softening; ≥1 cm dilatation + cervical softening; cervical length < 30 mm at endovaginal ultrasound. | FM, AF | PCR | GA was determined in all patients by routine ultrasound in the second trimester (16th–19th) weeks of gestation |  |  |  |  |  |  | 2/21 | 0.09524 |
| Holst | 2005 | Sweden | Within 12 hours of admission | SPTL: regular uterine contractions at a frequency of at least 2 uterine contractions/10 min for ≥ 30 min (confirmed by external tocometry) in combination with documented cervical ripening (≤2 cm length + ≥ 1 cm dilatation or ≤ 2 cm length + cervical softening or cervical length < 30 mm measured by vaginal ultrasound). | CF, AF | PCR | GA was determined by routine ultrasound in the second trimester (16th to 19th weeks of gestation) in all patients. |  |  | 1/50 | 0.0200 |  |  | 23/50 | 0.46000 |
| Kim | 2012 | Republic of Korea | NM | NM | AF | culture | NM |  |  | 1/132 | 0.0076 |  |  | 2/132 | 0.01515 |
| Lee | 2013 | Korea | NM | SPTL: presence of regular uterine contractions with a frequency of at least two every 10 min and cervical change before 37 completed weeks of gestation that required hospitalization. | AF | culture | NM |  |  | 0/237 | 0.0000 |  |  | 9/237 | 0.03797 |
| Koucký | 2016 | Czech Republic | NM | SPTL: birth before the 37th week of pregnancy | VS | PCR | The LMP and confirmed by the first trimester ultrasound scan |  |  |  |  |  |  | 17/29 | 0.58621 |
| Cox | 2016 | Northen Ireland | At delivery | NM | P | PCR | NM | 0/57 | 0.0000 | 0/57 | 0.0000 | 11/57 | 0.19298 | 2/57 | 0.03509 |
| Yoneda | 2017 | Japan | NM | SPTL: presence of regular uterine contractions (6 hour or more), cervical changes of more than 50% effaced, and dilatation of the cervix or shortening of the cervical length to less than 25 mm before 37 completed weeks of gestation | AF | PCR, culture and sequencing | LMP or ultrasound findings of the crown-rump length (CRL) size between 8 and 10 weeks |  |  |  |  | 9/105 | 0.08571 | 4/105 | 0.03810 |
| Choi | 2012 | Korea | Labor | NM | VF | PCR and culture | NM |  |  | 16/126 | 0.1270 |  |  | 79/129 | 0.62698 |
| Yoon | 1998 | Korea | Labor | SPTL was defined as the presence of regular uterine contractions with a frequency of at least two every 10 minutes and cervical change. | AF | culture | NM |  |  |  |  |  |  | 11/181 | 0.06077 |
| Romero | 1989 | USA | Labor | SPTL was defined as the presence of regular uterine contractions with a frequency of at least two every 10 minutes. | AF | culture | NM |  |  | 4/264 | 0.0152 |  |  | 6/264 | 0.02273 |
| Yoon | 2003 | Korea | GA <35week | NM | AF | PCR and culture | NM |  |  |  |  |  |  | 21/124 | 0.16935 |
| Cahill | 2005 | United Kingdom | After delivery | NM | FM | PCR | NM |  |  | 2/14 | 0.1429 |  |  |  |  |
| **PPROM** | | | | | | | | | | | | | | | |
| Averbuch | 1995 | Israel | NM | NM | AF | culture | NM |  |  | 1/90 | 0.0111 |  |  | 18/90 | 0.20000 |
| Jacobsson | 2003 | Sweden | Within 12 hours of admission | NM | AF | PCR | Routine ultrasound on 2nd trimester |  |  |  |  |  |  | 8/53 | 0.15094 |
| Perni | 2004 | USA | 2nd trimester | PPROM: rupture of the chorioamniotic membrane before the onset of labor at <37 weeks of gestation. | AF | PCR and ELISA | NM |  |  | 2/5 | 0.4000 |  |  | 3/5 | 0.60000 |
| Shim | 2005 | South Korea | NM | NM | AF | culture | Reviewing the clinical record |  |  | 1/121 | 0.0083 |  |  | 10/121 (pPROM) | 0.08264 |
| Nasution | 2007 | Malaysia | NM | PPROM: less than 37 weeks gestation | VF | PCR | NM |  |  | 2/40 | 0.0500 |  |  | 7/40 | 0.17500 |
| Kacerovsky | 2009 | Czech Republic | On admission before administration of corticosteroids, antibiotics or tocolytics. | NM | AF | PCR | LMP and confirmed by ultrasound measurement of a crown-rump length during the first trimester. |  |  |  |  | 2/29 | 0.068966 | 3/29 | 0.10345 |
| Kacerovsky | 2014 | Czech Republic | Delivery | PPROM: between GAs 24 and 36 | AF | PCR | First trimester fetal biometry |  |  | 3/29 | 0.1034 |  |  |  |  |
| Romero | 2014 | USA | Delivery | NM | AF | culture and PCR | LMP and confirmed by ultrasound examination, or by ultrasound examination alone if the sonographic determination of GA was not consistent with menstrual dating |  |  | 0/59 | 0.0000 | 2/59 | 0.033898 | 3/59 | 0.05085 |
| Musilova | 2017 | Czech Republic | NM | PPROM: leakage of AF prior to the onset of labor | AF | PCR and sequencing | First-trimester ultrasonography |  |  | 1/287 | 0.0035 |  |  |  |  |
| Musilova | 2017 | Czech Republic | NM | PPROM: leakage of AF prior to the onset of labor | AF | PCR and sequencing | First-trimester ultrasonography |  |  | 6/479 | 0.0125 |  |  |  |  |
| Jayaprakash | 2016 | Canada | between 24+0- and 33+6-weeks GA | Ruptured membranes were confirmed by evidence of VF pooling, a positive nitrazine test (elevated pH), and a positive ferning test | VF | PCR | NM |  |  |  |  | 14/36 | 0.388889 | 5/36 | 0.138889 |
| Gauthier | 1994 | USA | Admission at hospital for PPROM | NM | AF | culture | NM |  |  |  |  |  |  | 33/225 | 0.146667 |
| Olomu | 2009 | USA | At delivery | Women delivering between GA 23 to 27 6⁄7 weeks | chorion | culture | NM |  |  |  |  |  |  | NA | 0.35000 |

**Abbreviations:** AF: amniotic fluid, C: cervix; CF: cervical fluid, CS: cervical swab; CV: cervicovaginal sample; CVF: cervicovaginal fluid; FM: fetal membrane; GA: gestational age; LMP: last menstrual period; NM: not mentioned; NA: not available; P: placenta; PCR: polymerase chain reaction; VF: vaginal fluid; VS: vaginal swab
