# Supplementary material for: Genital Mycoplasmas and Biomarkers of Inflammation and Their Association With Spontaneous Preterm Birth and Preterm Prelabor Rupture of Membranes: A Systematic Review and Meta-Analysis
Source: Front Microbiol. 2022 Mar 30;13:859732. doi: 10.3389/fmicb.2022.859732 (PMC9006060; doi:10.3389/fmicb.2022.859732)
Supplement: Supplementary file 7 [file Table_6.docx]

**Supplementary Table 6.** Qualitative synthesis of case-control and cohort studies showing the association between genital Mycoplasma infections and SPTL, PTB, and PPROM patients.

| **First author** | **Year** | **Country** | **Time of screening** | **Definition of outcome (SPTL, PTB, PPROM)** | **Specimen Used** | **Method of Detection** | **Gestational age measurement** | **Odds Ratio** | | | |
| --- | --- | --- | --- | --- | --- | --- | --- | --- | --- | --- | --- |
|  |  |  |  |  |  |  |  | ***M. genitalium*** | ***M. hominis*** | ***U. parvum*** | ***U. urealyticum*** |
| **Preterm birth** | | | | | | | | | | | |
| Elliott | 1990 | Kenya | Case: enrolled within 24 h after birth/ Control: labor and delivery. All cultures were collected within 24 h of delivery and again at postpartum day 7 | Women who delivered a single livebirth neonate weighing ≤ 2500 g and in whom the clinical examination suggested a GA of ≤ 36 weeks | VF | culture | GA was estimated by the length of skin creases across the sole of foot, the presence of a palpable breast nodule, the morphology of the external auricle, and the presence of rugae on the scrotum |  | 1.31 [0.76, 2.24] |  | 1.03 [0.58, 1.82] |
| Holst | 1994 | Sweden | When the patient was admitted for being in labor | Delivery occurring between 34 and <37 weeks gestation and delivery occurring prior to 34 weeks gestation | VF | culture | Based on the LMP combined with ultrasonographic data |  | 1.55 [0.35, 6.82] |  | 0.66 [0.24, 1.83] |
| Alfa | 1995 | Canada | Day of delivery | NM | T, P, V | culture | Date of maternal LMP |  |  |  | 0.68 [0.35, 1.30] |
| Abele-Horn | 2009 | Germany | Time of admission (after 23 weeks of gestation had been completed) | Delivery before completed 37+0 weeks of pregnancy. SPTL: defined as regular uterine contractions (two or more in 10 minutes), or by labor that resulted in a PTB | VF, P | culture | Based on the LMP combined with ultrasonographic data |  |  |  | 3.31 [1.79, 6.12] |
| Yoon | 2001 | South Korea | mid-trimester | Delivery before 32 weeks of gestation | AF | PCR | NM |  |  |  | 40.52 [2.00, 821.05] |
| Povlsen | 2001 | Denmark | first antenatal visit for all samples and also at delivery for preterm cases | Delivery of <37 full gestational weeks | CF | PCR | NM |  |  |  | 1.02 [0.64, 1.65] |
| Labbé | 2002 | Guinea-Bissau | After delivery | Delivery of <37 full gestational weeks | C | PCR | Date of LMP and/or the uterine height | 1.37 [0.74, 2.53] |  |  |  |
| Harada | 2008 | Japan | First visit due to symptoms (preterm delivery) or at the onset of labor pains (full‐term delivery) | NM | VF | culture and PCR | NM |  |  |  | 2.44 [1.18, 5.03] |
| Hitti | 2010 | Peru | After delivery (within 48h) | Delivery at 20 to 36 weeks’ gestation | CVF | PCR | Newborn physical examination | 2.50 [1.27, 4.95] |  |  |  |
| Montenegro | 2019 | Colombia | After delivery | Delivery at <37 weeks | O, P | PCR | NM |  | 0.75 [0.07, 8.44] |  | 0.21 [0.01, 4.13] |
| McGregor | 1990 | USA | At approximately 24 weeks' gestation. | Birth before completion of 37 weeks' gestation. | VS | culture | GA was determined by overall "best" obstetric criteria that included history of LMP, clinical dating criteria (uterine size at first examination, GA at detection of fetal heart rate by fetoscope, growth of fundal height), and ultrasonographic measurements, as well as newborn examination |  | 5.73 [1.30, 25.26] |  | 2.92 [0.59, 14.41] |
| McDonald | 1992 | Australia | 22-28 weeks of gestation | Delivery before 37 completed weeks gestation | VS | culture | NM |  | 1.43 [0.71, 2.88] |  | 2.04 [1.40, 2.97] |
| Vogel | 2006 | Denmark | Range: 7 weeks and 4 days 23 weeks and 3 day | Spontaneous onset of birth before 37 weeks gestation | VF | culture | GA was based upon LMP confirmed by ultrasonographic measurements (biparietal diameter and femur length, week 18) |  |  |  |  |
| Donders | 2009 | Belgium | 9 - 16 weeks of pregnancy | < 36 weeks + 6 days | VF | culture | GA of the fetus was conﬁrmed by ultrasound before 16 weeks |  | 8.51 [2.69, 26.98] |  |  |
| Bohm | 2019 | Austria | 12 - 14 weeks of gestation | <37 weeks gestation | V | PCR | GA was determined based on the ultrasound scan in the first trimester of pregnancy. |  |  | 1.73 [1.34, 2.23] |  |
| Rittenschober-Bohm | 2018 | Austria | 12 - 14 weeks of gestation | Vaginal delivery or caesarean section after SPTL or PPROM | VS | PCR | GA was determined based on the ultrasound in the first trimester of pregnancy |  |  | 1.64 [1.29, 2.08] | 1.13 [0.69, 1.83] |
| Menard | 2010 | France | <34 weeks of pregnancy | Delivery at <37 weeks of pregnancy | VF | PCR | NM |  | 2.00 [0.26, 15.16] |  |  |
| Rodriguez | 2011 | Cuba | 16 – 20 weeks of gestation | Birth at 37 weeks of gestation proceeded by preterm labor with intact membranes. | AF | PCR and culture | NM |  |  | 2.09 [0.09, 46.38] | 2.09 [0.09, 46.38] |
| Averbach | 2013 | USA | Before 16 weeks of gestation | Delivery at more than 24 weeks but less than 37 weeks of gestation | C | PCR | GA was determined by the LMP and confirmed by ultrasound performed at 6 to 16 weeks of gestation | 1.28 [0.13, 12.64] |  |  |  |
| McDonald | 1994 | Australia | Between 22 and 28 weeks of gestation and again when they were admitted in spontaneous labor. | Delivery before 37 weeks of gestation | C | culture | GA determined by combination of LMP and first or second trimester ultrasonography |  | 10.36 [1.68, 63.81] |  | 1.78 [0.89, 3.57] |
| Payne | 2016 | Australia | Within the first or second trimester | Delivery at <37 weeks of pregnancy | VS | PCR | NM | 7.73 [1.27, 46.92] | 1.48 [0.31, 7.20] | 5.87 [1.56, 22.13] | 1.19 [0.25, 5.73] |
| Soromon | 2006 | Japan | <11 weeks of gestation | Delivery at 34 weeks of gestation | VF | PCR | GA was determined by a combination of the LMP and ultrasonographic evaluation. | 2.63 [0.15, 47.61] | 1.91 [0.63, 5.79] | 3.03 [1,10, 8.33] | 0.52 [0.07, 3.93] |
| Riikka | 2002 | Finland | Third trimester of pregnancy | NM | CF | PCR and culture | NM |  |  |  | 4.06 [1.33, 12.40] |
| Edwards | 2006 | USA | Between 23 and 32 weeks of gestation | Delivery at <37 weeks of pregnancy | VF | PCR | NM |  |  |  |  |
| **Preterm labor** | | | | | | | | | | | |
| Lamont | 1987 | England | Third trimester | NM | VF | culture | NM |  | 3.62 [0.77, 16.96] |  | 7.23 [2.61, 20.06] |
| Hillier | 1988 | USA | Second or third trimester | Two or more regular contractions every 10 minutes for 2 hours or more at GA of less than 37 weeks. | P | culture | Date of the LMP, clinical dating and ultrasonographic data |  | 2.65 [0.49, 14.26] |  | 2.45 [1.05, 5.76] |
| Holst | 1994 | Sweden | Labor (Time the patient was admitted for being in labor) | PTB was subdivided into delivery occurring between 34 and <37 weeks and delivery occurring prior to 34 weeks gestation | VF | culture | LMP combined with ultrasonographic data |  | 7.22 [0.86, 60.50] |  | 0.73 [0.31, 1.75] |
| Mitsunari | 2005 | Japan | First visit due to symptoms | Presence of regular uterine contractions with a frequency of at least one every 10min and cervical change | CF | PCR | NM |  |  |  | 7.90 [2.12, 29.49] |
| Gonzalez Bosquet | 2006 | Spain | Case: at the time of admission (24 and 34 weeks of gestation) and for control: during routine prenatal visits | Presence of cervical changes detected by the Bishop test and regular uterine contractions (a minimum of two contractions every 10 min) | C | culture | Early first-trimester ultrasonographic examination |  | 0.08 [0.00, 2.05] |  |  |
| Marconi | 2011 | Brazil | From 40 total samples, 15 were collected during c-section and the other 25 were not specified | Less than 37 weeks of gestation, presence of regular uterine contractions each 10 min or less and cervical effacement equal or superior to 50% confirmed by two observers and/or cervical dilatation of at least 2 cm | AF | PCR | Ultrasound before 20 weeks |  | 10.23 [1.12, 93.34] |  | 8.20 [0.40, 169.90] |
| Minkoff | 1984 | USA | First prenatal visit | Intact membranes who at <37 weeks' gestation demonstrated regular contractions (more than one contraction every 8 minutes) and either a change in the cervix during observation in the hospital or an already well-effaced (>75%) cervix or dilatation of >1cm | VF | culture | NM |  | 1.36 [0.65, 2.84] |  | 2.54 [1.05, 6.11] |
| McGregor | 1990 | USA | At approximately 24 weeks' gestation. | Regular uterine contractions before 37 weeks' gestation for which patients received treatment (e.g., parenteral or oral tocolytic treatment or intravenous hydration and bed rest, or contractions that resulted in preterm birth). | VF | culture | GA was determined by overall "best" obstetric criteria that included history of LMP, clinical dating criteria (uterine size at first examination, GA at detection of fetal heart rate by fetoscope, growth of fundal height), and ultrasonographic measurements, as well as newborn examination. |  | 2.06 [0.68, 6.24] |  | 1.53 [0.71, 3.31] |
| Toth | 1992 | UK | Labor | NM | C, V, P | culture | NM |  | 1.25 [0.12, 13.00] |  | 0.41 [0.14, 1.18] |
| Abele-Horn | 2009 | Germany | Time of admission (after 23 weeks of gestation had been completed) | Regular uterine contractions (two or more in 10 minutes), or by labor that resulted in a PTB | VF, P | culture | Based on the LMP combined with ultrasonographic data |  |  |  | 5.00 [2.25, 11.12] |
| **PPROM** | | | | | | | | | | | |
| Minkoff | 1984 | USA | First prenatal visit (13.8 ± 3.6 weeks' gestation) | Spontaneous rupture of the membranes prior to the onset of labor before the completion of 37 weeks of gestation | VF | culture | NM |  | 2.04 [1.01, 4.14] |  |  |
| Kacerovsky | 2009 | Czech Republic | Time of their admission | Rupture of membranes at gestation age between 24 and 36 weeks | CF | culture | NM |  | 2.11 [1.33, 3.36] |  |  |

**Abbreviations:** AF: amniotic fluid, C: cervix; CF: cervical fluid, CS: cervical swab; CV: cervicovaginal sample; CVF: cervicovaginal fluid; FM: fetal membrane; GA: gestational age; LMP: last menstrual period; NM: not mentioned; NA: not available; P: placenta; PCR: polymerase chain reaction; VF: vaginal fluid; VS: vaginal swab
