# Supplementary material for: Genital Mycoplasmas and Biomarkers of Inflammation and Their Association With Spontaneous Preterm Birth and Preterm Prelabor Rupture of Membranes: A Systematic Review and Meta-Analysis
Source: Front Microbiol. 2022 Mar 30;13:859732. doi: 10.3389/fmicb.2022.859732 (PMC9006060; doi:10.3389/fmicb.2022.859732)
Supplement: Supplementary file 10 [file Table_9.docx]

**Supplementary Table 9.** Subgroup analysis of the prevalence of genital mycoplasma among PTB, PTL, and PPROM based on the diagnostic methods used to detect genital mycoplasma.

| **Genital mycoplasma** | **Culture** | | **PCR** | | **Culture and PCR** | |
| --- | --- | --- | --- | --- | --- | --- |
|  | **Prevalence** | **95% CI** | **Prevalence** | **95% CI** | **Prevalence** | **95% CI** |
| **Preterm birth** | | | | | | |
| *M. genitalium* | 0.03 | -0.01 - 0.07 | 0.03 | 0.00 - 0.06 | - | - |
| *M. hominis* | 0.10 | -0.02 - 0.22 | 0.02 | -0.01 - 0.05 | 0.37 | 0.04 - 0.71 |
| *U. parvum* | 0.26 | -0.07 - 0.60 | 0.34 | 0.18 - 0.51 | - | - |
| *U. urealyticum* | 0.26 | 0.17 - 0.34 | 0.22 | -0.15 - 0.60 | 0.5 | 0.15 - 0.85 |
| **Preterm labor** | | | | | | |
| *M. genitalium* | - | - | 0.01 | -0.02 - 0.03 | - | - |
| *M. hominis* | 0.01 | 0.00 - 0.01 | 0.02 | 0.00 - 0.04 | 0.13 | 0.07 - 0.19 |
| *U. parvum* | - | - | 0.13 | 0.03 - 0.23 | - | - |
| *U. urealyticum* | 0.17 | 0.06 - 0.29 | 0.17 | 0.00 - 0.34 | 0.39 | -0.04 - 0.82 |
| **Preterm prelabor rupture of membrane** | | | | | | |
| *M. genitalium* | - | - | - | - | - | - |
| *M. hominis* | 0.05 | 0.005 - 0.10 | 0.01 | 0.00 - 0.02 | - | - |
| *U. parvum* | - | - | 0.15 | -0.06 - 0.36 | - | - |
| *U. urealyticum* | 0.28 | 0.194 - 0.38 | 0.12 | 0.07 - 0.18 | - | - |
