# Supplementary material for: Genital Mycoplasmas and Biomarkers of Inflammation and Their Association With Spontaneous Preterm Birth and Preterm Prelabor Rupture of Membranes: A Systematic Review and Meta-Analysis
Source: Front Microbiol. 2022 Mar 30;13:859732. doi: 10.3389/fmicb.2022.859732 (PMC9006060; doi:10.3389/fmicb.2022.859732)
Supplement: Supplementary file 11 [file Table_10.docx]

**Supplementary Table 10.** Subgroup analysis of the prevalence of genital mycoplasma among PTB, PTL, and PPROM based on the specimen used to detect genital mycoplasma.

| **Genital mycoplasma** | **Fetal samples** | | **Maternal samples** | |
| --- | --- | --- | --- | --- |
|  | **Prevalence** | **95% CI** | **Prevalence** | **95% CI** |
| **Preterm birth** | | | | |
| *M. genitalium* | 0.03 | -0.01 - 0.07 | 0.03 | 0.00 - 0.06 |
| *M. hominis* | 0.16 | -0.04 - 0.35 | 0.04 | 0.01 - 0.06 |
| *U. parvum* | 0.27 | -0.07 - 0.60 | 0.34 | 0.18 - 0.51 |
| *U. urealyticum* | 0.18 | 0.11 - 0.25 | 0.34 | 0.20 - 0.49 |
| **Preterm labor** | | | | |
| *M. genitalium* | 0.01 | -0.02 - 0.03 | - | - |
| *M. hominis* | 0.01 | 0.00 - 0.01 | 0.16 | 0.06 - 0.28 |
| *U. parvum* | 0.13 | 0.03 - 0.23 | - | - |
| *U. urealyticum* | 0.05 | 0.03 - 0.07 | 0.51 | 0.33 - 0.70 |
| **Preterm prelabor rupture of membrane** | | | | |
| *M. genitalium* | - | - | - | - |
| *M. hominis* | 0.01 | 0.01 - 0.02 | 0.09 | 0.03 - 0.15 |
| *U. parvum* | 0.04 | 0 - 0.08 | 0.39 | 0.23 - 0.55 |
| *U. urealyticum* | 0.23 | 0.16 - 0.29 | 0.38 | 0.14 - 0.62 |
