# Supplementary material for: Genital Mycoplasmas and Biomarkers of Inflammation and Their Association With Spontaneous Preterm Birth and Preterm Prelabor Rupture of Membranes: A Systematic Review and Meta-Analysis
Source: Front Microbiol. 2022 Mar 30;13:859732. doi: 10.3389/fmicb.2022.859732 (PMC9006060; doi:10.3389/fmicb.2022.859732)
Supplement: Supplementary file 12 [file Table_11.docx]

**Supplementary Table 11.** Qualitative synthesis of studies which reported polybacterial genital Mycoplasma infection during pregnancy and preterm birth.

| **Author** | **Year** | **Prevalence of *M. hominis* and *U. urealyticum* coinfection** | |
| --- | --- | --- | --- |
|  |  | **Raw Count** | **Proportion** |
| **Preterm Delivery** | | | |
| Goldenberg | 2008 | 18/351 | 0.05 |
| **Spontaneous Preterm Labor** | | | |
| Gravett | 1986 | 0/54 | 0 |
| Watts | 1992 | 1/105 | 0.01 |
| Romero | 1992 | 0/52 | 0 |
| Horowitz | 1995 | 9/47 | 0.19 |
| Holst | 2005 | 1/50 | 0.02 |
| Kim | 2012 | 0/132 | 0 |
| Lee | 2013 | 9/237 | 0.04 |
| **PPROM** | | | |
| Romero | 1992 | 2/22 | 0.09 |
| Grattard | 1995 | 33/208 | 0.16 |
| Horowitz | 1995 | 5/34 | 0.15 |
